# Lanthanide-Connecting and Lone-Electron-Pair Active Trigonal-Pyramidal-AsO_3_ Inducing Nanosized Poly(polyoxotungstate) Aggregates and Their Anticancer Activities

**DOI:** 10.1038/srep26406

**Published:** 2016-05-19

**Authors:** Jun-Wei Zhao, Hai-Lou Li, Xing Ma, Zhigang Xie, Li-Juan Chen, Yongsheng Zhu

**Affiliations:** 1 Henan Key Laboratory of Polyoxometalate Chemistry, Institute of Molecule and Crystal Engineering, College of Chemistry and Chemical Engineering, Henan University, Kaifeng, 475004, China; 2 State Key Laboratory of Polymer Physics and Chemistry, Changchun Institute of Applied Chemistry, Chinese Academy of Sciences, Changchun 130022, China; 3 Department of Physics, Nanyang Normal University, Nanyang 473061, China

## Abstract

By virtue of the stereochemical effect of the lone-electron pair located on the trigonal-pyramidal-AsO_3_ groups and the one-pot self-assembly strategy in the conventional aqueous solution, a series of novel lanthanide-bridging and lone-electron-pair active trigonal-pyramidal-AsO_3_ inducing nanosized poly(polyoxotungstate) aggregates [H_2_N(CH_3_)_2_]_6_ Na_24_H_16_{[Ln_10_W_16_(H_2_O)_30_O_50_](B-α-AsW_9_O_33_)_8_}·97H_2_O [Ln = Eu^III^ (**1**), Sm^III^ (**2**), Gd^III^ (**3**), Tb^III^ (**4**), Dy^III^ (**5**), Ho^III^ (**6**), Er^III^ (**7**), Tm^III^ (**8**)] were prepared and further characterized by elemental analyses, IR spectra, UV spectra, thermogravimetric (TG) analyses and single-crystal X-ray diffraction. The most remarkable structural feature is that the polyanionic skeleton of {[Ln_10_W_16_(H_2_O)_30_O_50_](B-α-AsW_9_O_33_)_8_}^46−^ is constructed from eight trivacant Keggin [B-α-AsW_9_O_33_]^9−^ fragments through ten Ln centers and sixteen bridging W atoms in the participation of fifty extraneous oxygen atoms. Notably, **4** and **8** can be stable in the aqueous solution not only for eight days but also in the range of pH = 3.9–7.5. Moreover, the cytotoxicity tests of **4** and **8** toward human cervical cancer (HeLa) cells, human breast cancer (MCF–7) cells and mouse fibroblast (L929) cells were performed by the 3-(4,5-cimethylthiazol-2-yl)-2,5-diphenyl tetrazolium bromide (MTT) assay and the cell apoptosis processes were characterized by calcein AM/PI staining experiments, annexin V-FITC/PI staining experiments and morphological changes.

Since the first polyoxometalate (POM) (NH_4_)_3_PMo_12_O_40_ was discovered in 1826^1^, POM chemistry as a fast-growing domain has been known for almost two centuries. As a class of fascinating versatile metal-oxide clusters, POMs have drawn considerable attraction because of their unrivalled structural diversities coupled with potential applications in diverse fields such as catalysis, medicine and materials science^2^,^3^,^4^. With the rapid progress of nanoscience and nanotechnology in recent years, the designed synthesis and assembly of large ploy(POM) nanosized materials have gradually emerged as a current forefront of chemistry due to the scientific importance for probing structures and bonding fundamentals, the requirement for extending the application range of new materials, and unique chemical and physical properties derived from their nanodimensions^5^,^6^. Hitherto, some key synthetic details on transition-metal (TM) encapsulated nanosized ploy(POM)s (TMENPs) have been well established and some typical TMENPs have been prepared^7^,^8^,^9^,^10^,^11^,^12^,^13^. However, relevant reports on lanthanide (Ln) encapsulated nanosized ploy(POM)s (LENPs) are very limited. Since the pioneer work involving compact water-soluble LENP [As^III^_12_Ce^III^_16_(H_2_O)_36_W_148_O_524_]^76−^ was reported by Pope in 1997^14^, some novel LENPs have been continuously discovered such as [(PEu_2_W_10_O_38_)_4_(W_3_O_14_)]^30− ^15, [K⊂{Eu(H_2_O)_2_(α-AsW_9_O_33_)}_6_]^35− ^16, [Cs⊂{Eu(H_2_O)_2_(α- AsW_9_O_33_)}_4_]^23− ^16, [Ce_20_Ge_10_W_100_O_376_(OH)_4_(H_2_O)_30_]^56− ^17, [Gd_8_As_12_W_124_O_432_(H_2_O)_22_]^60− ^18, [Tb_8_(pic)_6_(H_2_O)_22_(B-β- AsW_8_O_30_)_4_(WO_2_(pic))_6_]^12− ^19, and [{(XO_3_)W_10_O_34_}_8_{Ce_8_(H_2_O)_20_}(WO_2_)_4_(W_4_O_12_)]^48−^ (X = Se^IV^, Te^IV^)^20^. Evidently, the majority of above-mentioned LENPs were synthesized in the multi-stepwise program by reaction of prefabricated lacunary POM precursors with Ln ions. Recently, the one-step reaction strategy has been gradually developed as an important synthetic approach for preparing nanosized ploy(POM)s and the potential of this strategy has been exemplified by Cronin’s reports on a library of remarkable gigantic ploy(polyoxtungstate)s^5^,^11^,^21^. The investigations on this approach used for the self-assembly reaction of simple tungstates and arsenite with Ln cations are underdeveloped to date, which provides us an excellent opportunity to explore this domain.

On one hand, the application of inorganic chemistry in medicine has become a burgeoning area of research with the development of cross-disciplinarity and cisplatin as a representative example has been applied to the treatment of cancer^22^,^23^. In the past several decades, it has proved that POMs can generically show broad spectrum antiviral, antitumor and antibacterial activities^24^,^25^. For example, (NH_4_)_17_Na[NaSb_9_W_21_O_86_]·nH_2_O (HPA-23) as the first POM antiviral agent was used for clinical trials by Jasmin *et al*. in 1973^26^. The *in-vivo* inhibitory effects on Meth A sarcoma and MM-46 adenocarcinoma of (NH_3_Pri)_6_[Mo_7_O_24_]·3H_2_O (PM-8) were reported by Yamase and co-workers in 1988^27^. Keggin-type polyoxotungstates against methicillin-resistant Staphylococcus aureus were investigated by Yamase *et al*. in 1999^28^. Subsequently, Hill *et al*. addressed the Nb^V^-containing Wells-Dawson POM HIV-1 protease inhibitors and conducted theoretical, binding, and kinetics studies of the POM/HIV-1 protease interactions^24^. In 2010, Dolbecq’s group studied the *in-vitro* tumor-cell-killing activities of a series of bisphosphonate functionalized polyoxomolybdate clusters^25^. In 2013, Zhou and collaborators probed the inhibitory effect of a trivacant Keggin tungstobismuthate on human gastric adenocarcinoma SGC-7901 cells^29^. In 2014, Wei *et al*. evaluated the antiproliferation performance of an amantadine-substituted hexamolybdate toward MCF–7 cells^30^. These bioactivities are intimately involved in their versatilities including oxygen-rich surfaces, controllable sizes, shapes, compositions, charge density, solubility, polarity, redox potential, nucleophilicity and acid strength^24^,^29^. However, to date, biological investigations on LENPs remain less developed in comparison with abundant TM-containing POM species, which mainly originates from the great difficulty in obtaining Ln-containing POMs in the past because the combination of lacunary POM fragments with Ln ions usually result in the amorphous precipitates. On the other hand, it is well known that currently cancer is a crucial universal disease with the high morbidity and the mortality that leads to the deaths of over 7 million people per annum, and is forecasted to turn into a more serious problem in the following twenty years^31^,^32^. Recently, developing water-soluble and biocompatible nanosized anticancer drugs has attracted significant interest with the wide application of nanotechnology. However, exploration and discovery of novel benign anticancer drugs still remains a great challenge and requires the long-term persistence in medicinal chemistry.

Under this research background, we have launched explorations on the lone-electron-pair trigonal-pyramidal-XO_3_ inducing syntheses of LENPs (X = As^III^, Sb^III^, Bi^III^, Se^IV^, Te^IV^) and further examine the anticancer activities on the base of the following ideas: (a) the stereochemical effect of the lone-electron pairs located on trigonal pyramidal XO_3_ groups encapsulated in POM lattices can to some degree hinder the closure of cage-like POM intermediates and thus favors to induce or direct the self-assembly of large ploy(POM)s; (b) due to the multiple coordination requirements and high oxophilicity, Ln electrophiles can function as excellent connectors to capture *in-situ*-generated POM intermediates, giving rise to novel LENPs; (c) the synergistic interactions between bifunctional active POM segments (as H^+^/e^−^ reservoirs) and Ln electrophiles can improve and enhance the medical activities and related properties of the desired products; (d) the acidic aqueous reaction environments can efficaciously decrease the precipitation probability of Ln elements and are beneficial to the one-step reaction and elaborative combination of simple tungstates, XO_3_-containing initial materials and Ln salts to create novel ploy(polyoxtungstate) aggregates. Herein, we report a class of novel lone-electron-pair active trigonal-pyramidal-AsO_3_ inducing LENPs [H_2_N(CH_3_)_2_]_6_Na_24_H_16_{[Ln_10_W_16_(H_2_O)_30_O_50_](B-α- AsW_9_O_33_)_8_}·97H_2_O [Ln = Eu^III^ (**1**), Sm^III^ (**2)**, Gd^III^ (**3)**, Tb^III^ (**4)**, Dy^III^ (**5)**, Ho^III^ (**6)**, Er^III^ (**7)**, Tm^III^ (**8)**]. The viability tests of **4** and **8** against HeLa and MCF–7 cells have been examined by the MTT assay and the cell apoptosis processes have been studied by calcein AM/PI staining experiments, annexin V-FITC/PI staining experiments and morphological changes.

## Results and Discussion

### Structural description

**Figure 1:**
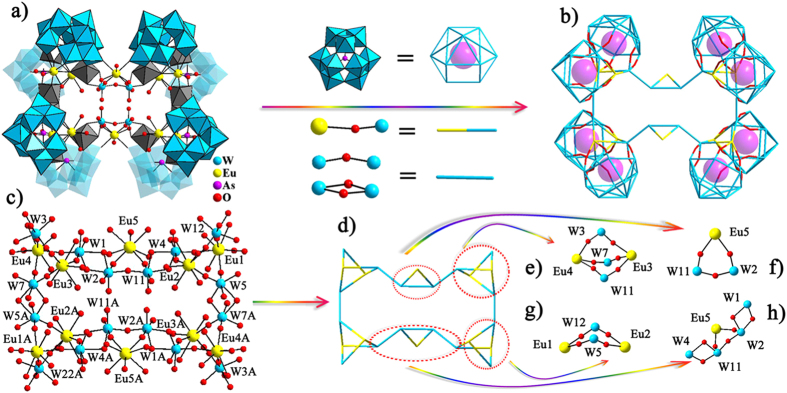
(**a**) The centrosymmetric polyanionic framework of {[Eu_10_W_16_(H_2_O)_30_O_50_](B-α-AsW_9_O_33_)_8_}^46−^ (**1a**) with the size of ca. 26.3 × 29.4 Å. (**b**) The simplified mode of **1a** with an aesthetic skeleton. (**c**) The rectangular [Eu_10_W_16_(H_2_O)_30_O_50_]^26+^ cluster core. (**d**) The simplified mode of [Eu_10_W_16_(H_2_O)_30_O_50_]^26+^ cluster core. (**e**) The {W_3_Eu_2_} subunit. (**f** ) The bridging {W_2_Eu} subunit. (**g**) The {W_2_Eu_2_} subunit. (**h**) The interesting semilunar {W_4_Eu} subunit.

The good phase purity of **1–8** is verified by the consistency of powder X-ray diffraction patterns (PXRD) of the as-prepared samples of **1–8** with the simulated XRD patterns derived from single-crystal structural analyses (Figure S1). X-ray diffraction structural analysis indicates that **1**–**8** are isomorphous and crystallize in the triclinic space group *P*−1. Thus, the structure of **1** is herein discussed as an example below. The centrosymmetric octameric polyoxoanionic framework {[Eu_10_W_16_(H_2_O)_30_O_50_](B-α-AsW_9_O_33_)_8_}^46−^ (**1a**) with about 26.3 × 29.4 Å in size is a fresh blood of LENP family (Fig. 1a). As demonstrated in Fig. 1b, eight trivacant Keggin-type [B-α-AsW_9_O_33_]^9−^ moieties jointly encapsulate a central rectangular [Eu_10_W_16_(H_2_O)_30_O_50_]^26+^ cluster core (Figure S2) to form the basic skeleton of **1a**, which are arranged in an well-proportioned distribution on the periphery of the central core. The trivacant [B-α-AsW_9_O_33_]^9−^ moiety is composed of a central AsO_3_ unit [As−O: 1.765(18)–1.800(16) Å] and three corner-sharing W_3_O_13_ traids [W−O: 1.678(18)–2.443(15) Å]. Obviously, the formation of the stable trivacant [B-α-AsW_9_O_33_]^9−^ moiety with six exposed surface oxygen atoms in the trivacant position is benefited from the inducing effect of the lone-electron-pair active trigonal-pyramidal-AsO_3_ group^14^,^22^. The intriguing rectangular [Eu_10_W_16_(H_2_O)_30_O_50_]^26+^ cluster core (Fig. 1c,d) can be viewed as a combination of four {W_3_Eu_2_} (namely W1W3W7Eu3Eu4, W4W5W12Eu1Eu2, W1AW3AW7AEu3AEu4A, W4AW5W12AEu1AEu2A) and two {W_2_Eu_1_} (namely W2W11Eu5, W2AW11 AEu5A) segments (Fig. 1e,f). In each {W_3_Eu_2_} segment, two eight-coordinate Eu^III^ centers are combined together by three W^VI^ centers via three Eu−O−W−O−Eu linkers. All the Eu^III^ cations in the {W_3_Eu_2_} segments reside in the distorted square antiprismatic geometries defined by two oxygen atoms from the lacunary position of one [B-α-AsW_9_O_33_]^9−^ moiety [Eu−O: 2.328(13)–2.410(17) Å] (Table S1), three oxygen atoms from three octahedral {WO_6_} groups [Eu−O: 2.284(16)–2.411(15) Å] and three water ligands [Eu−O: 2.382(18)–2.527(16) Å] (Figure S3a–h). In the triangle {W_2_Eu_1_} segment, the seven-coordinate mono-capped trigonal prismatic geometry of the Eu5^III^ cation is finished by two terminal oxygen atoms from two [B-α-AsW_9_O_33_]^9−^ moieties [Eu−O: 2.33(2)–2.375(17) Å], two oxygen atoms from two octahedral {WO_6_} groups [Eu−O: 2.404(15)–2.404(16) Å] and three water ligands [Eu−O: 2.40(2)–2.57(3) Å], which is distinct from the square antiprismatic geometry of the eight-coordinate Eu^III^ cations in the {W_3_Eu_2_} segments (Figure S3i,j).

**Figure 2:**
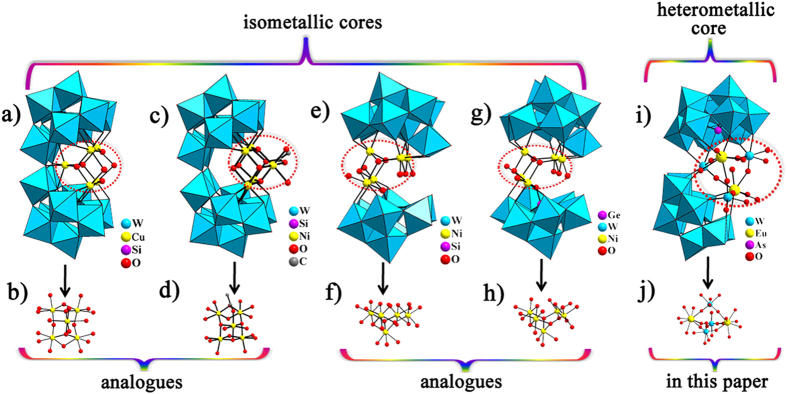
(**a**) View of [Cu_5_(OH)_4_(H_2_O)_2_(A-α-SiW_9_O_33_)_2_]^10−^. (**b**) View of the monometallic pentanuclear [Cu_5_(OH)_4_(H_2_O)_2_]^6+^ core. (**c**) View of {[Ni_5_(OH)_3_(H_2_O)_4_(CH_3_CO_2_)][Si_2_W_18_O_66_]}^6−^. (**d**) View of the monometallic pentanuclear [Ni_5_(OH)_3_(H_2_O)_4_ (CH_3_CO_2_)]^6+^ core. (**e**) View of [H_2_{Ni_5_(H_2_O)_5_(OH)_3_(x-SiW_9_ O_34_) (β-SiW_8_O_31_)}_2_]^24−^. (**f**) View of the monometallic pentanuclear [Ni_5_(H_2_O)_5_(OH)_3_]^+7^ core. (**g**) View of [Ni_5_(OH)_4_(H_2_O)_4_(β-GeW_9_O_34_)(β-GeW_8_O_30_(OH))]^13−^. (**h**) View of the monometallic pentanuclear [Ni_5_(OH)_4_(H_2_O)_4_]^6+^ core. (**i**) The skeleton of **1c**. (**j**) View of the heterometallic pentanuclear [Eu_2_(H_2_O)_6_W_3_O_10_]^4+^ core.

Each {W_3_Eu_2_} segment in **1a** is combined with two trivacant Keggin [B-α-AsW_9_O_33_]^9−^ moieties via eight W−O−W and four W−O−Eu linkers to generate a pentanuclear heterometallic sandwich-type primary unit {Eu_2_(H_2_O)_6_W_3_O_10_[B-α- AsW_9_O_33_]_2_}^14−^ (**1c**) (Figs 2a and S4). Hitherto, several pentanuclear sandwich-type POMs have been reported (Fig. 2). In 2005, Kortz *et al*. communicated a penta-Cu^II^ sandwiched tungstosilicate [Cu_5_(OH)_4_(H_2_O)_2_(A-α-SiW_9_O_33_)_2_]^10−^ (Fig. 2a) that can be viewed as an open Wells–Dawson anion chelating a central [Cu_5_(OH)_4_(H_2_O)_2_]^6+^ core (Fig. 2b) in the vacant site^33^. In 2013, a penta-Ni^II^ substituted tungstosilicate hybrid {[Ni_5_(OH)_3_(H_2_O)_4_(CH_3_CO_2_)][Si_2_W_18_O_66_]}^6−^ (Fig. 2c) with the similar open Wells–Dawson anion skeleton and a novel [Ni_5_(OH)_3_(H_2_O)_4_(CH_3_CO_2_)]^6+^ hybrid core (Fig. 2d) was obtained by Song and co-workers^34^. In 2007, a penta-Ni^II^ substituted tungstosilicate [H_2_{Ni_5_(H_2_O)_5_(OH)_3_(x-SiW_9_O_34_)(β- SiW_8_O_31_)}_2_]^24−^ (Fig. 2e) with mixed [x-SiW_9_O_34_]^10−^ and [α-SiW_8_O_31_]^10−^ building blocks connected by a [Ni_5_(H_2_O)_5_(OH)_3_]^7+^ group (Fig. 2f) was isolated by Wang *et al*.^35^. Analogously, a penta-Ni^II^ containing germanotungstate [Ni_5_(OH)_4_(H_2_O)_4_(β- GeW_9_O_34_)(β-GeW_8_O_30_(OH))]^13−^ (Fig. 2g) was also synthesized by Kortz and collaborators, which can be viewed as a combination of a trilacunary [β-GeW_9_O_34_]^10−^ and a tetralacunary [β-GeW_8_O_30_(OH)]^9−^ linked by a [Ni_5_(OH)_4_(H_2_O)_4_]^6+^ core (Fig. 2h)^36^. The remarkable differences between **1c** (Fig. 2i) and the above-mentioned four pentanuclear sandwich-type POMs lie in two aspects: a) **1c** own a heterometallic pentanuclear central core (Fig. 2j) whereas others have the isometallic pentanuclear cores, b) **1c** was prepared from the one-pot reaction of simple materials of Na_2_WO_4_·2H_2_O and NaAsO_2_ while others were made by the prefabricated precursors such as K_10_[A-α-SiW_9_O_34_], Na_10_[α-SiW_9_O_34_]·18H_2_O, K_8_[γ-SiW_10_O_36_] or K_8_[γ-GeW_10_O_36_]·6H_2_O. Upon the remove of two Eu^III^ centers from **1c**, an interesting lacunary [As_2_W_21_O_76_]^20−^ dimeric unit can be formed (Figure S5b) and is constructed from two [B-α-AsW_9_O_33_]^9−^ moieties bridged by one {WO_6_} octahedron and two pendent {WO_6_} octahedra, which is apparently different from the previously reported [As_2_W_21_O_69_(H_2_O)]^6−^ precursor (Figure S5c)^37^, in which two [B-α-AsW_9_O_33_]^9−^ segments are symmetrically located on both sides of a central plane defined by three W = O groups. When two pendent {WO_6_} octahedra are removed from the lacunary [As_2_W_21_O_76_]^20−^ dimeric unit, the remaining [As_2_W_19_O_68_]^16−^ fragment (Figure S5d) is distinct from the symmetric [As_2_W_19_O_67_(H_2_O)]^14−^ polyoxoanion (Figure S5e)^38^, the distorted structural characteristic of the [As_2_W_19_O_68_]^16−^ fragment should be highlighted and is mainly derived from the incorporation of two Eu^III^ ions and two pendent {WO_6_} octahedra.

**Figure 3:**
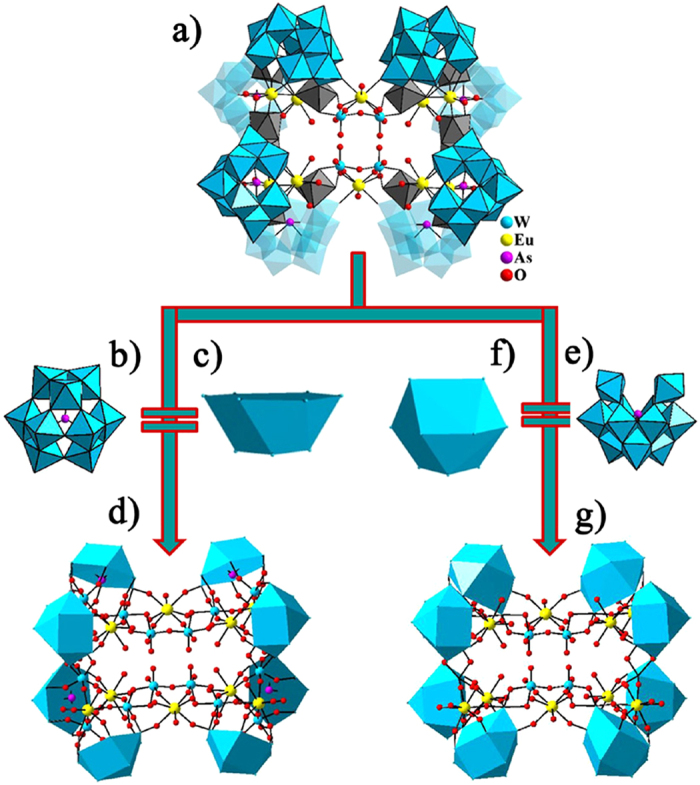
(**a**) The molecular structural unit of **1a**. (**b**) The [B-α-AsW_9_O_33_]^9−^ segment. (**c**) The simplified polyhedron of the [B-α-AsW_9_O_33_]^9−^ segment. (**d**) The simplified model of **1a**. (**e**) The [B-α-AsW_11_O_41_]^13−^ segment. (**f**) The simplified polyhedron of the [B-α-AsW_11_O_41_]^13−^ segment. (**g**) The simplified mode of **1a**.

Notably, two **1c** primary units can be connected together by the bridging {W_2_Eu_1_} segment via four W−O−W and two W−O−Eu bridges, giving rise to the secondary unit (the asymmetric unit) {[Eu_5_W_8_(H_2_O)_15_O_25_](B-α-AsW_9_O_33_)_4_}^23−^ (**1b**) (Figure S6). And then, two **1b** secondary units are symmetrically related through the inversion center with atomic coordinate of (0, 0, 1) generating the tertiary unit (the molecular structural unit) {[Eu_10_W_16_(H_2_O)_30_O_50_](B-α-AsW_9_O_33_)_8_}^46−^ (**1a**) (Figure S6). Upon a careful observation of the asymmetry unit (Figure S7a), two types of heterometallic sandwich-type segments in **1b** are almost mirror-symmetric to each other (Figure S7b). Interestingly, after the removal of Eu^III^ ions, the two remaining [As_2_W_21_O_76_]^20−^ units in **1b** still keep this mirror-symmetry (Figure S7c). However, the centrosymmetry of the whole polyanionic framework leads to the racemization of **1a**, and thus **1** can’t show the chirality and the circular dichroism optical activity. Above all, the skeleton of the giant tungsten cluster of **1a** with the omission of ten Eu^III^ centers not only demonstrates the existence of eighty-eight W centers, but also highlights the structure-stabilizing effect of Eu^III^ ions in the formation of the giant tungsten cluster (Figures S8b,c). In addition, the rectangle [Eu_10_W_16_(H_2_O)_30_O_50_]^26+^ cluster core can also be divided into four {W_2_Eu_2_} (namely, W3W7Eu3Eu4, W5W12Eu1Eu2, W3AW7AEu3AEu4A, W5AW12AEu1AEu2A) and two {W_4_Eu} (namely, W1W2W11 W4Eu5, W1AW2AW11AW4AEu5A) segments (Fig. 1g,h). In each {W_2_Eu_2_} subunit, two W centers are bridged together by two square antiprismatic Eu^III^ cations via two W−O−Eu−O−W linkers. Each {W_4_Eu} segment consists of a semilunar {W_4_} group with a supporting Eu^III^ cation, in which two edging-sharing {W_2_} moieties are integrated by sharing an oxygen atom and a Eu^III^ cation. This semilunar {W_4_} group is entirely distinct from the rhombic {W_4_} group in [enH_2_]_2_[Ni(H_2_O)_4_]_2_ [Ni(en)_2_]_2_[Ni(en)]_2_{[(α- AsW_6_O_26_)Ni_6_(OH)_2_(H_2_O)_3_(en)(B-α-AsW_9_O_34_)]_2_[W_4_O_16_][Ni_3_(H_2_O)_2_(en)]_2_}·16H_2_O (Figure S9a,b)^39^, the square {W_4_} group in K_32_Na_16_[{(SeO_3_)W_10_O_34_}_8_{Ce_8_(H_2_O)_20_}(WO_2_)_4_(W_4_O_12_)]·81H_2_O (Figure S9c)^20^, and the S-shaped {W_4_} group in K_12_Na_22_[{(SeO_3_)W_10_O_34_}_8_{Ce_8_(H_2_O)_20_}(WO_2_)_4_{(W_4_O_6_)Ce_4_(H_2_O)_14_(SeO_3_)_4_(NO_3_)_2_}]·79H_2_O (Figure S9d)^20^. On the other hand, when the [B-α-AsW_9_O_33_]^9−^ units are simplified as polyhedra (Fig. 3b,c), the simplified model of **1a** is shown in Fig. 3d. When the [B-α-AsW_11_O_41_]^13−^ units are simplified as polyhedra (Fig. 3e,f), the simplified model of **1a** is displayed in Fig. 3g. In addition, the 3-D arrangements of **1a** along three a, b, c axes are shown in Figure S10.

### Aqueous solution stability and anticancer activities

**Figure 4:**
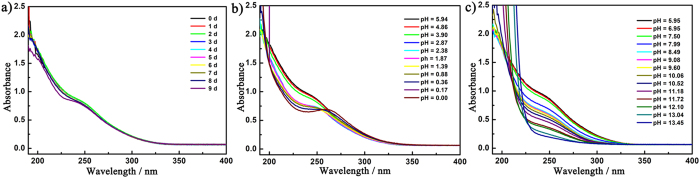
(**a**) The UV spectral evolution of **4** with time. (**b**) The UV spectral evolution of **4** in acidic direction. (**c**) The UV spectral evolution of **4** in alkaline direction.

**Figure 5:**
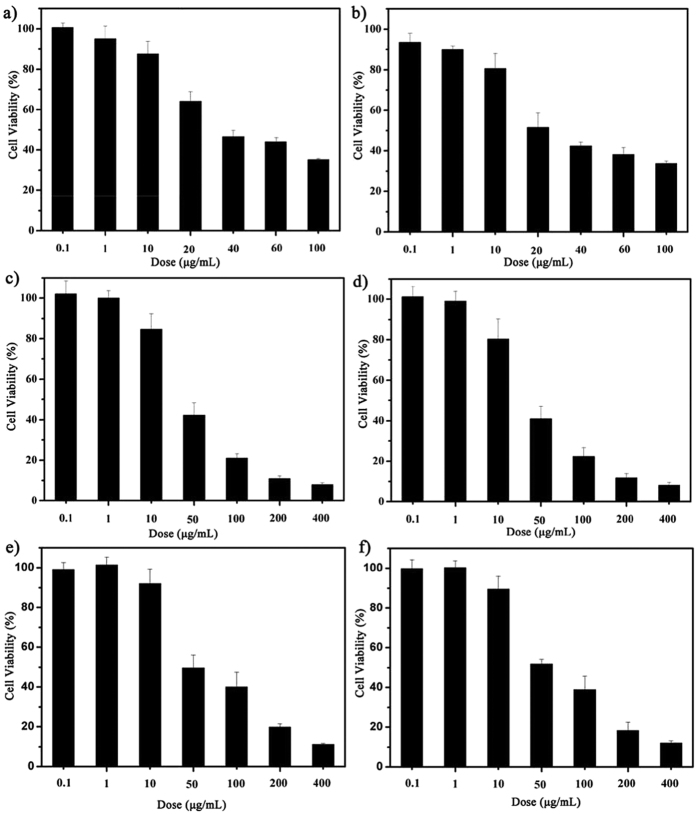
(**a**) Cytotoxicity resulting from a 48 h **4** treatment of HeLa cells. (**b**) Cytotoxicity resulting from a 48 h **8** treatment of HeLa cells. (**c**) Cytotoxicity resulting from a 48 h **4** treatment of MCF–7 cells. (**d**) Cytotoxicity resulting from a 48 h **8** treatment of MCF–7 cells. (**e**) Cytotoxicity resulting from a 48 h **4** treatment of L929 cells. (**f** ) Cytotoxicity resulting from a 48 h **8** treatment of L929 cells.

**Figure 6:**
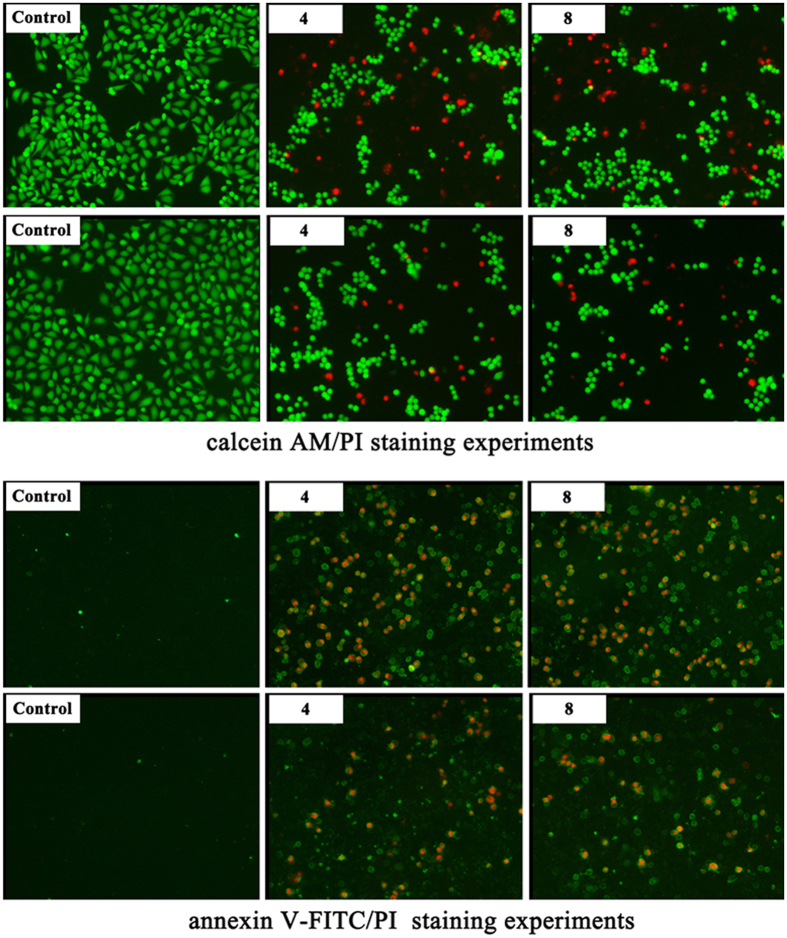
Top: the fluorescence microscopy images of 4 and 8 against HeLa cells with concentration of 1 mg/mL for 6 h that is typically determined with calcein AM/PI staining and the fluorescence microscopy images of 4 and 8 against MCF–7 cells with concentration of 1 mg/mL for 6 h that is typically determined with calcein AM/PI staining. Bottom: the fluorescence microscopy images of HeLa cells incubated by **4** and **8** with concentration of 1 mg/mL for 8 h and the fluorescence microscopy images of MCF–7 cells incubated by **4** and **8** with concentration of 1 mg/mL for 8 h.

**Figure 7:**
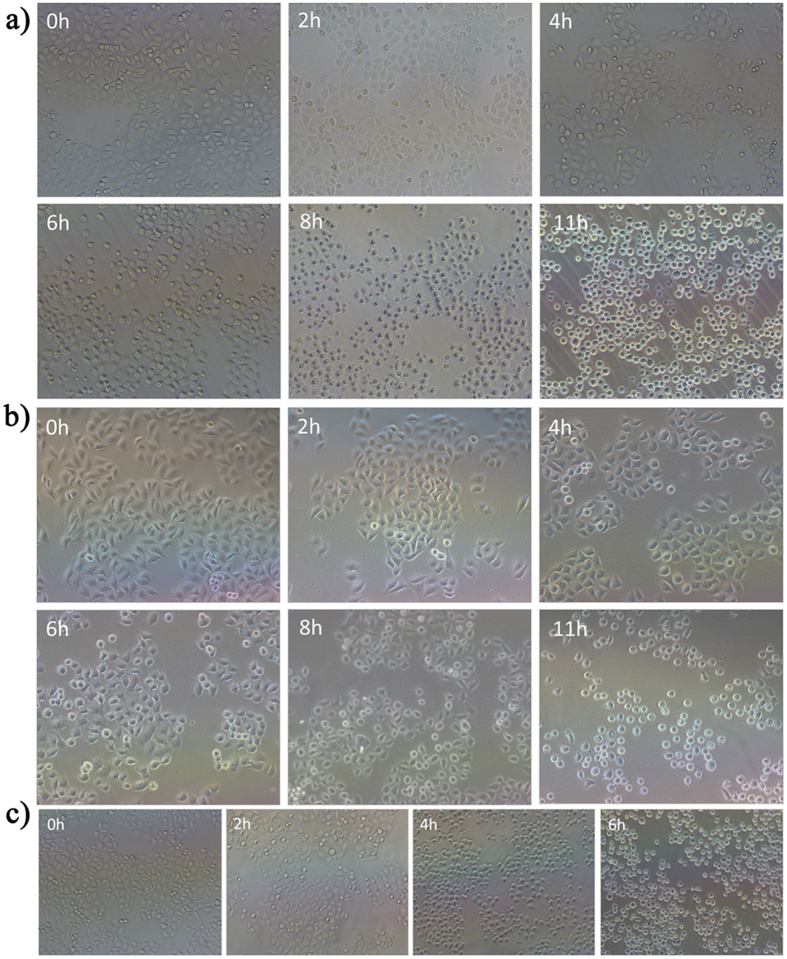
Morphological changes of HeLa cells incubated by (**a**) **4**, (**b**) **8** and (**c**) K_14_[As_2_W_19_O_67_(H_2_O)] with concentrations of 1 mg/mL.

In order to study the aqueous solution stability of **1–8**, the UV spectra of **4** and **8** in the aqueous solution as representatives have been investigated in the range of 190–400 nm at room temperature. Both UV spectra exhibit a strong absorption band peak at ca. 194 nm (**4**) and ca. 195 (**8**) that can be ascribed to the O_t_ → W pπ–dπ charge-transfer transitions and a weaker absorption band at ca. 248 nm (**4**) and ca. 247 nm (**8**) that can be attributed to the O_b(c)_ → W pπ–dπ charge-transfer transitions (Figure S11)^40^. It is noteworthy that the UV spectra of **4** and **8** almost remain unchanged at room temperature for eight days (Figs 4a and S12a), which preliminarily imply that **4** and **8** are stable in aqueous solution within eight days and provide a necessity for performing their biological evaluation. To further probe the dependence of **4** and **8** on the pH variation in aqueous solution, the UV spectra of **4** and **8** in acidic and alkaline regions have been measured. The pH values are adjusted by using diluted H_2_SO_4_ and NaOH. It should be noted that the initial pH values of **4** and **8** dissolved in aqueous solution are about 5.94 and 5.88, respectively. Experimental results indicate that the UV spectrum of **4** has no conspicuous change in the pH scope of 3.90–7.50. However, the intensity of the O_b(c)_ → W absorption band decreases and a new broad centered at 260 nm comes to appear when the pH is gradually lower than 3.90 (Fig. 4b) whereas the O_b(c)_ → W absorption band gradually becomes weaker until disappearing and the O_t_ → W absorption band become more and more stronger upon the pH being higher than 7.50 (Fig. 4c). Therefore, a conclusion could be drawn that the pH stable range of **4** in aqueous solution is about 3.9–7.5. Similarly, **8** is stable in the pH scope of ca. 3.9–7.4 (Figures S12–c). This fact suggests that **4** and **8** can be stable in human blood environment (pH = 7.3–7.5), which provides a clear guidance that **4** and **8** can be utilized potentially *in vivo* for exerting their antitumor activities. As illustrated in Figure S13, the presence of the O_t_ → W and O_b(c)_ → W absorption bands in the UV spectra of **4** and **8** dissolved in the 0.3 mmol/L PBS (phosphate buffered saline) or the 0.3 mmol/L PBS containing 0.3% FBS (fetal bovine serum) further supports that **4** and **8** can maintain their structural integrity in the blood environment. To examine the potential of **4** and **8** as antitumor agents, **4** and **8** were then used for evaluating their tumor-cell-killing ability *in vitro*. The human cervical cancer (HeLa) cells, human breast cancer (MCF–7) cells were exposed to **4** and **8** in different doses for 48 h. The cytotoxicity was evaluated using MTT assay and test results are illustrated in Fig. 5a–d. The cytotoxic tests of **4** and **8** against HeLa and MCF–7 cells indicate the dose-dependent behavior. The cell viability of HeLa and MCF–7 cells decreases below 40% as the concentration of **4** or **8** increases to 100 μg/mL (35.17% of **4** against HeLa; 33.74% of **8** against HeLa; 21.12% of **4** against MCF–7; 22.38% of **8** against MCF–7). The IC_50_ values (the concentration of a compound that produces 50% cell death) of **4** against HeLa and MCF–7 cells are 40.05 μg/mL and 40.32 μg/mL, respectively, while the IC_50_ values of **8** against HeLa and MCF–7 cells are 24.76 μg/mL and 37.01 μg/mL, respectively (Table S2). In comparison with IC_50_ values of **4** and **8** against normal L929 cells (59.68 μg/mL and 58.04 μg/mL, respectively) (Fig. 5e,f, Table S2), it can be concluded that **4** and **8** exhibit the higher cytotoxicity against HeLa and MCF–7 cells than against normal L929 cells, indicating that **4** and **8** behave as considerable anticancer activities in killing HeLa and MCF–7 cells. It is well known that arsenic compounds have been extensively exploited as anti-proliferative drugs and can induce complete remission of the cancer patients with relapsed^41^,^42^. Hence, the IC_50_ values of K_14_[As_2_W_19_O_67_(H_2_O)] against HeLa and MCF–7 cells have been also tested as control (Table S2). The IC_50_ values of K_14_[As_2_W_19_O_67_(H_2_O)] against HeLa and MCF–7 cells are 27.56 μg/mL and 32.00 μg/mL, respectively. Obviously, the cytotoxicity of **4** or **8** is lower than that of K_14_[As_2_W_19_O_67_(H_2_O)], but the reason is not clear for us right now. For the cytotoxicity of nanomedicines, several parameters could affect their cell viability, including the size, shape and stability. As reported in our previous work, the IC50 values of cisplatin toward HeLa and MCF–7 cells are 1.03 and 2.63 μg/mL, respectively^43^. Considering the IC50 value, **4** or **8** are less toxic than cisplatin. The living and dead cancer cells can be observed by calcein AM/PI staining experiments. It can be clearly seen from the fluorescence microscopy images (The upper of Fig. 6) that the control cells emit green fluorescence, which signifies that they are alive. However, most HeLa and MCF–7 cells incubated by **4** and **8** with the concentration of 1 mg/mL after 6 h exhibit the fluorescence color change from green to red, which indicate that they have been dead^44^. Numerous studies have shown that apoptosis is a typical form for chemotherapy drug-induced cell death. For example, cisplatin and its generation analogues can induce DNA damage and then arrest the cancer cells at the G2/M phase of the whole cell cycle^45^,^46^. Arsenic trioxide can trigger apoptosis and autophagy of leukemia cell lines^47^,^48^. For the purpose of verifying that apoptosis induces the cell death for HeLa and MCF–7 cells, the widely used fluorescent staining of Annexin V-FITC together with PI were used to **4** and **8**. Generally, after staining a cell population with Annexin V-FITC and PI, apoptotic cells show green fluorescence, dead cells or necrosis cells emit red fluorescence, and live cells exhibit little or no fluorescence. Thereby, Annexin V-FITC/PI staining method can distinguish apoptosis cells and necrosis cells. As shown in the bottom of Fig. 6, the images of control groups don’t display fluorescence, indicating that cells are live cells other than necrosis cells. It can be apparently seen from the fluorescence microscopy images of HeLa and MCF–7 cells incubated by **4** and **8** with the concentration of 1 mg/mL after 8 h that a large number of apoptotic cells can be observed and red fluorescence overlaps with green fluorescence, which clearly demonstrating that **4** and **8** induce the apoptosis of HeLa and MCF–7 cells and further cause the death of cells. On the other hand, apoptosis is a mode of programmed cell death and is usually accompanied by a series of cell morphological changes of such as pyknosis, chromatin condensation, nuclear condensation, nuclear fragmentation, cell surface blebbing and so on^49^. Therefore, in order to further confirm the apoptosis process, morphological changes of HeLa and MCF–7 cells incubated by **4**, **8** and K_14_[As_2_W_19_O_67_(H_2_O)] with concentration of 1 mg/mL are examined using optical microscope. As shown in Fig. 7a, HeLa cells incubated in the medium of **4** begin to shrink and become round as time goes on. After 11 h, almost all the HeLa cells have shrinked and become round. These results demonstrate that apoptosis induces the death of HeLa cells in the presence of **4**. Similar results of **8** and K_14_[As_2_W_19_O_67_(H_2_O)] toward HeLa cells and **4**, **8** and K_14_[As_2_W_19_O_67_(H_2_O)] toward MCF–7 cells can be seen from Figs 7b,c and S14.

### IR spectra and thermostability

IR spectra of **1**–**8** haven been conducted on a Nicolet 170 SXFT–IR spectrometer in the range of 400–4000 cm^−1^ with KBr pellets. Due to the existence of the trivacant Keggin [B-α-AsW_9_O_33_]^9−^ fragments in the skeletons of **1**–**8**, IR spectra in the low wave-number region all exhibit four similar characteristic terminal *ν*(W−O_t_), *ν*(As−O_a_), corner-sharing *ν*(W−O_b_) and edge-sharing *ν*(W−O_c_) asymmetric stretching vibration modes, which are seen at 948–952 cm^−1^, 862–869 cm^−1^, 783–786 cm^−1^ and 707–713 cm^−1^, respectively (Figure S15)^50^,^51^. In their high wave-number region, an obvious broad band in the range of 3438–3446 cm^−1^ corresponds to the ν(O−H) stretching mode of lattice or coordination water molecules. Additionally, those signals appearing at 3157–3168 cm^−1^ and 2800–2813 cm^−1^ can be ascribed to the *ν*(N−H) and *ν*(C−H) stretching vibration modes whereas the resonances observed at 1625–1631 cm^−1^ and 1463–1467 cm^−1^ can be assigned to the *δ*(N−H) and *δ*(C−H) bending vibrations, which suggest the existence of dimethylamine components in **1**–**8**. However, because of the predominant ionic interactions between trivacant Keggin [B-α-AsW_9_O_33_]^9−^ fragments and Ln^3+^ ions, the Ln−O stretching vibration bands can’t be observed in the IR spectra.

**Figure 8:**
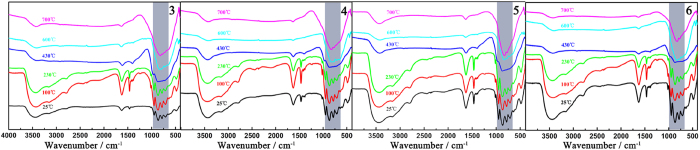
The VTIR spectra of **3–6** with the similar evolutional trend.

The thermostability of **3–6** has been also probed by multiply techniques including TG analyses, variable temperature powder X-ray diffraction (VTPXRD) patterns and variable temperature IR (VTIR) spectra. First of all, the TG analyses of **1–8** have been examined on the pure crystalline samples under the flowing nitrogen atmosphere in the temperature range of 25–900 °C with the heating rate of 10 °C min^−1^ (Figure S16). Obviously, the TG curves of **1–8** can be divided into three steps. The first weight loss of 6.35% (calcd. 6.61%) for **1**, 6.66% (calcd. 6.61%) for **2**, 6.52% (calcd. 6.59%) for **3**, 6.47% (calcd. 6.59%) for **4**, 6.55% (calcd. 6.58%) for **5**, 6.29% (calcd. 6.57%) for **6**, 6.28% (calcd. 6.57%) for **7** and 6.46% (calcd. 6.56%) for **8** between 25 to 200 °C correspond to the release of ninety-seven lattice water molecules. Another weight loss weight loss of 4.01% (calcd. 3.81%) for **1**, 3.75% (calcd. 3.82%) for **2**, 3.76% (calcd. 3.81%) for **3**, 4.08% (calcd. 3.80%) for **4**, 3.58% (calcd. 3.80%) for **5**, 4.09% (calcd. 3.80%) for **6**, 3.85% (calcd. 3.79%) for **7** and 4.02% (calcd. 3.79%) for **8** between 200 to 650 °C can be ascribed to the liberation of thirty coordinate water molecules, the dehydration of twenty-two protons and the release of six dimethylamine molecules. After 650 °C, a gradual weight loss of 2.54% (calcd. 2.99%) for **1**, 2.62% (calcd. 2.99%) for **2**, 2.59% (calcd. 2.99%) for **3**, 3.21% (calcd. 2.99%) for **4**, 2.59% (calcd. 2.98%) for **5**, 3.00% (calcd. 2.98%) for **6**, 3.13% (calcd. 2.98%) for **7** and 2.51% (calcd. 2.97%) for **8** until 900 °C may be attributed to the sublimation of four As_2_O_3_. Based on the TG results, the VTIR spectra of **3–6** as representatives were also measured at 25, 100, 230, 430, 600 and 700 °C and display the similar evolutional trend (Fig. 8). It is very clear that their characteristic vibration bands remain unchanged as temperature rises from 25 to 230 °C, which indicate the main skeletons of **3–6** are stable in this temperature region apart from the removal of some lattice water molecules. This result coincides well with the first weight loss of the TG curve. However, as temperature continues to increase to 430 °C, not only *ν*(W−O_t_), *ν*(As−O_a_), *ν*(W−O_b_) and *ν*(W−O_c_) vibration bands in the low-wavelength region gradually disappear, but also the *ν*(N−H) and *ν*(C−H) vibration modes become unobvious. These phenomena provide the evidence that the skeletons of **3–6** are undergoing the thermal decomposition process. When temperature reaches 600 °C, the *ν*(N−H) and *ν*(C−H) vibration signals have vanished, indicating the liberation of dimethylamine groups. On the other hand, the VTPXRD patterns of **3–6** further support TG and VTIR results. As illustrated in Figure S17, all diffraction peaks almost retain unchangeable before 100 °C, being indicative of the good crystallinity of **3–6**, which further illustrate that the structures of **3–6** are almost no change except for the loss of some lattice water molecules. Upon heating to ca. 430 °C, most of characteristic diffraction peaks gradually disappear, which principally originates from the fact that the crystalline samples of **3–6** have been efflorescent and led to the very bad crystallinity of **3–6** when all the lattice water molecules and some coordinate water molecules are removed away from of **3–6**. This fact can be also confirmed by the results of TG analyses and VTIR spectra. After 600 °C, the occurrence of some new diffraction peaks in the PXRD patterns at 600 and 700 °C reveals that new decomposition phases come to emerge, which demonstrates that the dehydration of protons and the sublimation of part As_2_O_3_ result in the decomposition of polyoxoanionic skeletons of **3–6**. This observation is also consolidated by the apparent distinction of IR spectra at 600 and 700 °C from those at 25, 100, 230 and 430 °C.

In summary, a series of novel nanosized LENPs [H_2_N(CH_3_)_2_]_6_Na_24_H_16_{[Ln_10_W_16_(H_2_O)_30_O_50_](B-α-AsW_9_O_33_)_8_}·97H_2_O [Ln = Eu^III^ (**1**), Sm^III^ (**2)**, Gd^III^ (**3)**, Tb^III^ (**4)**, Dy^III^ (**5)**, Ho^III^ (**6)**, Er^III^ (**7)**, Tm^III^ (**8)**] have been successfully isolated based on the stereochemical effect of the lone-electron pairs located on trigonal pyramidal AsO_3_ groups located on polyoxtungstate fragments and the connection role of Ln cations. Intriguingly, the multi-Ln incorporated octameric framework {[Ln_10_W_16_(H_2_O)_30_O_50_](B-α-AsW_9_O_33_)_8_}^46−^ consists of eight trivacant Keggin [B-α-AsW_9_O_33_]^9−^ fragments linked by ten Ln ions and sixteen bridging W atoms in the presence of fifty extraneous oxygen atoms. Moreover, the aqueous solution stability and thermostability of some representatives have been investigated. Furthermore, the cytotoxicity tests of **4** and **8** toward HeLa, MCF–7 and L929 cells have been examined by the MTT assay and the cell apoptosis processes have been characterized by calcein AM/PI staining experiments, annexin V-FITC/PI staining experiments and morphological changes. This finding opens the door to the research on medical activities of multi-Ln incorporated POMs and expands the research domain of POM chemistry. Our following work will be concentrated on expanding the designed syntheses and pharmaceutical activity evaluation of much more high nuclear LENPs (X = As^III^, Sb^III^, Bi^III^, Se^IV^, Te^IV^). Emphasis will put on investigating cancer cell apoptosis process and apoptosis mechanism.

## Methods

### Materials

All the reagents were purchased commercially and used without further purification.

### Preparation of [H_2_N(CH_3_)_2_]_6_Na_24_H_16_{[Eu_10_W_16_(H_2_O)_30_O_50_](B-α-AsW_9_O_33_)_8_}·97H_2_O (1)

Na_2_WO_4_·2H_2_O (1.400 g, 4.240 mmol) and dimethylamine hydrochloride (0.502 g, 6.156 mmol) were dissolved in water (20 mL) under stirring and NaAsO_2_ (0.5 mL, 1 moL·L^−1^) was added. After the pH of the resulting solution was adjusted to 4.0 by using hydrochloric acid (6.0 moL·L^−1^), Eu(NO_3_)_3_·6H_2_O (0.198 g, 0.444 mmol) was then added and the pH was again adjusted to 4.0. After stirring for 30 min, the solution was filtered and left at room temperature. Slow evaporation of the filtrate resulted in colorless prism crystals of **1** for several weeks. Yield: 0.30 g (25.5% based on Eu(NO_3_)_3_·6H_2_O). Elemental analysis calcd. (%) for C_12_H_318_As_8_ Eu_10_N_6_Na_24_ O_441_W_88_: C 0.54, H 1.21, N 0.32, Na 2.08, As 2.26, W 61.12, Eu 5.74; found: C 0.63, H 1.44, N 0.41, Na 2.17, As 2.19, W 60.88, Eu 5.82; IR (KBr): *v* = 3446 (s), 3168(s), 2813(m), 1629(m), 1463(w), 1396(w), 1253(w), 1018(w), 952(s), 867(s), 784(s), 707(w), 638(m), 601(m), 486(m) cm^−1^ (Figure S16).

### Preparation of [H_2_N(CH_3_)_2_]_6_Na_24_H_16_{[Sm_10_W_16_(H_2_O)_30_O_50_](B-α-AsW_9_O_33_)_8_}·97H_2_O (2)

The synthesis of **2** is similar to **1** with using Sm(NO_3_)_3_·6H_2_O (0.203 g, 0.457 mmol) instead of Eu(NO_3_)_3_·6H_2_O. Light yellow prism crystals of **2** were obtained for several weeks. Yield: 0.28 g (23.2% based on Sm(NO_3_)_3_·6H_2_O). Elemental analysis calcd (%) for C_12_H_318_As_8_Sm_10_N_6_Na_24_O_441_W_88_: C 0.54, H 1.21, N 0.32, Na 2.09, As 2.27, W 61.19, Sm 5.69; found: C 0.65, H 1.40, N 0.43, Na 2.21, As 2.21, W 60.95, Sm 5.73; IR (KBr): *v* = 3440 (s), 3159(s), 2800(m), 1627m), 1467(w), 1384(w), 1245(w), 1020(w), 950(s), 865(s), 783(s), 709(w), 634(m), 597(m), 482(m) cm^−1^ (Figure S16).

### Preparation of [H_2_N(CH_3_)_2_]_6_Na_24_H_16_{[Gd_10_W_16_(H_2_O)_30_O_50_](B-α-AsW_9_O_33_)_8_}·97H_2_O (3)

The synthesis of **3** is similar to **1** with using Gd(NO_3_)_3_·6H_2_O (0.201 g, 0.445 mmol) instead of Eu(NO_3_)_3_·6H_2_O. Colorless prism crystals of **3** were obtained for several weeks. Yield: 0.32 g (26.8% based on Gd(NO_3_)_3_·6H_2_O). Elemental analysis calcd (%) for C_12_H_318_As_8_Gd_10_N_6_Na_24_O_441_W_88_: C 0.54, H 1.21, N 0.32, Na 2.08, As 2.26, W 61.04, Gd 5.93; found: C 0.66, H 1.43, N 0.44, Na 2.17, As 2.20, W 60.57, Gd 5.80; IR (KBr): *v* = 3440 (s), 3163(s), 2804(m), 1629m), 1467(w), 1392(w), 1253(w), 1024(w), 950(s), 862(s), 784(s), 709(w), 644(m), 595(m), 491(m) cm^−1^ (Figure S16).

### Preparation of [H_2_N(CH_3_)_2_]_6_Na_24_H_16_{[Tb_10_W_16_(H_2_O)_30_O_50_](B-α-AsW_9_O_33_)_8_}·97H_2_O (4)

The synthesis of **4** is similar to **1** with using Tb(NO_3_)_3_·6H_2_O (0.199 g, 0.439 mmol) instead of Eu(NO_3_)_3_·6H_2_O. Colorless prism crystals of **4** were obtained for several weeks. Yield: 0.35 g (30.1% based on Tb(NO_3_)_3_·6H_2_O). Elemental analysis calcd (%) for C_12_H_318_As_8_Tb_10_N_6_Na_24_O_441_W_88_: C 0.54, H 1.21, N 0.32, Na 2.08, As 2.26, W 61.00, Tb 5.99; found: C 0.63, H 1.46, N 0.46, Na 2.19, As 2.18, W 60.87, Tb 6.13; IR (KBr): *v* = 3438 (s), 3163(s), 2810(m), 1633(m), 1467(w), 1386(w), 1239(w), 1022(w), 950(s), 867(s), 786(s), 711(w), 636(m), 597(m), 480(m) cm^−1^ (Figure S16).

### Preparation of [H_2_N(CH_3_)_2_]_6_Na_24_H_16_{[Dy_10_W_16_(H_2_O)_30_O_50_](B-α-AsW_9_O_33_)_8_}·97H_2_O (5)

The synthesis of **5** is similar to **1** with using Dy(NO_3_)_3_·6H_2_O (0.202 g, 0.442 mmol) instead of Eu(NO_3_)_3_·6H_2_O. Colorless prism crystals of **5** were obtained for several weeks. Yield: 0.32 g (27.2% based on Dy(NO_3_)_3_·6H_2_O). Elemental analysis calcd (%) for C_12_H_318_As_8_Dy_10_N_6_Na_24_O_441_W_88_: C 0.54, H 1.21, N 0.32, Na 2.08, As 2.26, W 60.91, Dy 6.12; found: C 0.62, H 1.40, N 0.45, Na 1.94, As 2.19, W 61.15, Dy 6.23; IR (KBr): *v* = 3440 (s), 3163(s), 2812(m), 1631(m), 1465(w), 1413(w), 1238(w), 1020(w), 952(s), 869(s), 786(s), 709(w), 638(m), 590(m), 482(m) cm^−1^ (Figure S16).

### Preparation of [H_2_N(CH_3_)_2_]_6_Na_24_H_16_{[Ho_10_W_16_(H_2_O)_30_O_50_](B-α-AsW_9_O_33_)_8_}·97H_2_O (6)

The synthesis of **6** is similar to **1** with using Ho(NO_3_)_3_·6H_2_O (0.200 g, 0.436 mmol) instead of Eu(NO_3_)_3_·6H_2_O. Light yellow prism crystals of **6** were obtained for several weeks. Yield: 0.38 g (32.8% based on Ho(NO_3_)_3_·6H_2_O). Elemental analysis calcd (%) for C_12_H_318_As_8_Ho_10_N_6_Na_24_O_441_W_88_: C 0.54, H 1.21, N 0.32, Na 2.08, As 2.25, W 60.86, Ho 6.20; found: C 0.64, H 1.41, N 0.39, Na 1.90, As 2.13, W 60.32, Ho 6.34; IR (KBr): *v* = 3446 (s), 3166(s), 2804(m), 1629(m), 1467(w), 1382(w), 1244(w), 1020(w), 948(s), 869(s), 784(s), 713(w), 644(m), 590(m), 489(m) cm^−1^ (Figure S16).

### Preparation of [H_2_N(CH_3_)_2_]_6_Na_24_H_16_{[Er_10_W_16_(H_2_O)_30_O_50_](B-α-AsW_9_O_33_)_8_}·97H_2_O (7)

The synthesis of **7** is similar to **1** with using Er(NO_3_)_3_·6H_2_O (0.201 g, 0.436 mmol) instead of Eu(NO_3_)_3_·6 H_2_O. Light pink prism crystals of **7** were obtained for several weeks. Yield: 0.36 g (31.0% based on Er(NO_3_)_3_·6H_2_O). Elemental analysis calcd (%) for C_12_H_318_As_8_Er_10_N_6_Na_24_O_441_W_88_: C 0.54, H 1.20, N 0.32, Na 2.07, As 2.25, W 60.80, Er 6.29; found: C 0.65, H 1.42, N 0.45, Na 1.92, As 2.11, W 60.02, Er 6.41; IR (KBr): *v* = 3446 (s), 3157(s), 2800(m), 1625(m), 1465(w), 1390(w), 1232(w), 1024(w), 950(s), 867(s), 783(s), 713(w), 646(m), 595(m), 482(m) cm^−1^ (Figure S16).

### Preparation of [H_2_N(CH_3_)_2_]_6_Na_24_H_16_{[Tm_10_W_16_(H_2_O)_30_O_50_](B-α-AsW_9_O_33_)_8_}·97H_2_O (8)

The synthesis of **8** is similar to **1** with using Tm(NO_3_)_3_·6H_2_O (0.204 g, 0.441 mmol) instead of Eu(NO_3_)_3_·6H_2_O. Colorless prism crystals of **8** were obtained for several weeks. Yield: 0.32 g (27.2% based on Tm(NO_3_)_3_·6H_2_O). Elemental analysis calcd (%) for C_12_H_318_As_8_Tm_10_N_6_Na_24_O_441_W_88_: C 0.54, H 1.20, N 0.32, Na 2.07, As 2.25, W 60.77, Tm 6.34; found: C 0.67, H 1.44, N 0.45, Na 1.96, As 2.15, W 60.91, Tm 6.45; IR (KBr): *v* = 3440 (s), 3157(s), 2804(m), 1629(m), 1465(w), 1406(w), 1238(w), 1020(w), 952(s), 865(s), 784(s), 711(w), 640(m), 584(m), 489(m) cm^−1^ (Figure S16).

### Single-crystal X-ray diffraction

Good-quality single crystals for **1–8** were carefully chosen from their mother liquids under the optical microscope and sealed in a capillary. Their diffraction data were collected on a Bruker Apex II diffractometer with the graphite monochromated Mo Kα radiation (*λ* = 0.71073 Å) at 296(2) K. Intensity data were corrected by Lorentz and polarization effect and empirical absorption on the base of the multi-scan technique. Their structures were solved by direct methods. The heavy atoms were located using the SHELXTL–97 program package^52^,^53^, and the remaining atoms were found from successive full-matrix least-squares refinements on *F*^2^ and Fourier syntheses. Those H atoms attached to C and N atoms were added in idealized geometrical positions. No H atoms linking to H_2_O molecules were found from the difference Fourier map. The non-H atoms were refined anisotropically except for some O, C, N atoms and H_2_O molecules. Solvent accessible voids are observed in the check cif reports of **1–8**, indicating that some highly disordered water molecules that can’t be found from the weak residual electron peaks may exist in their structures. We tried to locate and refine them, but we failed. Finally, according to the results of elemental analyses and TG measurements, seventy-six water molecules were directly added to each molecular formula. This phenomenon is very common in POM chemistry^54^. Crystallographic data and structural refinement parameters for **1–8** are listed in Table S3. CCDC–1421345 (**1**), 1421346 (**2**), 1421347 (**3**), 1421348 (**4**), 1421349 (**5**), 1421350 (**6**), 1421351 (**7**) and 1421352 (**8**) contain the supplementary crystallographic data for this paper. These data can be also obtained free of charge from The Cambridge Crystallographic Data Centre via www.ccdc.cam.ac.uk/data_request/cif.

### Elemental analyses

Elemental analyses (C, H, N) were performed using a Perkin–Elmer 240C elemental analyzer. Inductively coupled plasma atomic emission spectrometry (ICP-AES) was performed on a Perkin-Elmer Optima 2000 ICP-AES spectrometer.

### IR spectra

IR spectra were recorded from a powdered sample pelletized with KBr on a Nicolet 170 SXFT–IR spectrometer in the range of 400–4000 m^−1^.

### TG analyses

TG analyses were measured under a N_2_ atmosphere on a Mettler–Toledo TGA/SDTA 851^e^ instrument with a heating rate of 10 °C·min^−1^.

### PXRD

PXRD measurements were performed on a Bruker D8 Advance XRD diffractometer with Cu Kα radiation (*λ* = 1.54056 Å).

### UV spectra

UV spectra were obtained on a HITACHI U–4100 UV–Vis–NIR spectrometer at room temperature.

### Cell culture

HeLa, MCF–7 and L929 cell lines were grown in Dulbecco’s modified Eagle’s medium (DMEM) supplemented with 10% heat-inactivated fetal bovine serum (FBS). The cells were cultured at 37 °C under 5% CO_2_ atmosphere with the culture medium replaced once every day.

### MTT experiments

Cells harvested in a logarithmic growth phase were seeded in 96-well plates at a density of 105 cells per well and incubated in DMEM for 24 h. The medium was then replaced by **4** and **8** at various concentrations. The incubation was continued for 48 h. Then, 20 μL of MTT solution in phosphate buffered saline (PBS) with the concentration of 5 mg/mL was added and the plates were incubated for another 4 h at 37 °C, followed by removal of the culture medium containing MTT and addition of 150 μL of DMSO to each well to dissolve the formazan crystals formed. Finally, the plates were shaken for 5 min, and the absorbance of formazan product was measured at 490 nm by a microplate reader.

### Optical microscope observation

The cells were observed with an optical microscope (Nikon Eclipse Ti, Optical Apparatus Co., Ardmore, PA, USA).

## Additional Information

**How to cite this article**: Zhao, J.-W. *et al*. Lanthanide-Connecting and Lone-Electron-Pair Active Trigonal-Pyramidal-AsO_3_ Inducing Nanosized Poly(polyoxotungstate) Aggregates and Their Anticancer Activities. *Sci. Rep.* **6**, 26406; doi: 10.1038/srep26406 (2016).

## Supplementary Material

Supplementary Information
