# Supplementary material for: Lanthanide-Connecting and Lone-Electron-Pair Active Trigonal-Pyramidal-AsO3 Inducing Nanosized Poly(polyoxotungstate) Aggregates and Their Anticancer Activities
Source: Sci Rep. 2016 May 19;6:26406. doi: 10.1038/srep26406 (PMC4872259; doi:10.1038/srep26406)
Supplement: Supplementary Information [file srep26406-s1.doc]

# *Supporting Information*

**Lanthanide-Connecting and Lone-Electron-Pair Active Trigonal-Pyramidal-AsO_3_ Inducing Nanosized Poly(polyoxotungstate) Aggregates and Their Anticancer Activities**

**Jun-Wei Zhao^1^,*, Hai-Lou Li^1^, Xing Ma^1^, Zhigang Xie^2^,*, Li-Juan Chen^1^,*, and Yongsheng Zhu^3^,***

^1^Henan Key Laboratory of Polyoxometalate Chemistry, Institute of Molecule and Crystal Engineering, College of Chemistry and Chemical Engineering, Henan University, Kaifeng, 475004, China, E-mail: zhaojunwei@henu.edu.cn.

^2^State Key Laboratory of Polymer Physics and Chemistry, Changchun Institute of Applied Chemistry, Chinese Academy of Sciences, Changchun 130022, China, E-mail: xiez@ciac.ac.cn.

^3^Department of Physics, Nanyang Normal University, Nanyang 473061, China, E-mail: yongshengzhu0001@163.com

**Figure** **S1** (a) The experimental PXRD and simulated XRD patterns of the as-prepared sample of **1**. (b) The experimental PXRD and simulated XRD patterns of the as-prepared sample of **2**. (c) The experimental PXRD and simulated XRD patterns of the as-prepared sample of **3**. (d) The experimental PXRD and simulated XRD patterns of the as-prepared sample of **4**. (e) The experimental PXRD and simulated XRD patterns of the as-prepared sample of **5**. (f) The experimental PXRD and simulated XRD patterns of the as-prepared sample of **6**. (g) The experimental PXRD and simulated XRD patterns of the as-prepared sample of 7. (h) The experimental PXRD and simulated XRD patterns of the as-prepared sample of **8**.

**Figure S2** (*a*) The inorganic multi-dentate [B-α-AsW_9_O_33_]^9–^ ligand. (*b*) The rectangular [Eu_10_W_16_(H_2_O)_30_O_50_]^26+^ cluster core. (*c*) The representative chelating mode between two inorganic multi-dentate [B-α-AsW_9_O_33_]^9–^ ligands and the center core.

**Figure S3** (*a*-*h*) The eight-coordinate square antiprism geometries of Eu^III^ cations in the {W_3_Eu_2_} segments with the highlighted different sources of oxygen atoms. (*i*-*j*) The seven-coordinate monocapped trigonal prism geometry of the Eu^III^ cation in the {W_2_Eu} segment with the highlighted different sources of oxygen atoms. (The blue bonds link the oxygen atoms from water ligands, the green bonds link the bridging oxygen atoms from [B-α-AsW_9_O_33_]^9–^ units and the purple bonds link the oxygen atoms from other the octahedral W centers)

**Figure S4** The structural disassembly of **1a** in which the primary unit is the pentanuclear heterometallic sandwich-type unit {Eu_2_(H_2_O)_6_W_3_O_10_[B-α-AsW_9_O_33_]_2_}^14–^, the secondary unit is the asymmetric unit {[Eu_5_W_8_(H_2_O)_15_O_25_](B-α-AsW_9_O_33_)_4_}^23–^ and the tertiary unit is the molecular structural unit {[Eu_10_W_16_(H_2_O)_30_O_50_](B-α-AsW_9_O_33_)_8_}^46–^.

**Figure S5** (*a*) The skeleton of the primary unit {Eu_2_(H_2_O)_6_W_3_O_10_[B-α-AsW_9_O_33_]_2_}^14–^. (*b*) View of the interesting dimeric [As_2_W_21_O_76_]^20–^ unit from the primary unit. (*c*) The structure of the previously reported [As_2_W_21_O_69_(H_2_O)]^6–^ precursor. (*d*) The distorted [As_2_W_19_O_68_]^16–^ fragment. (*e*) The [As_2_W_19_O_67_(H_2_O)]^14–^ polyoxoanion.

**Figure** **S6** (*a*) and (*b*) The skeleton of the pentanuclear heterometallic sandwich-type unit {Eu_2_(H_2_O)_6_W_3_O_10_[B-α-AsW_9_ O_33_]_2_}^14–^. (*c*) The bridging {W_2_Eu_1_} segment. (*d*) View of the asymmetric unit {[Eu_5_W_8_(H_2_O)_15_O_25_](B-α-AsW_9_O_33_)_4_}^23–^. (*e*) The skeleton of the symmetric unit {[Eu_10_W_16_(H_2_O)_30_ O_50_](B-α-AsW_9_O_33_)_8_}^46–^.

**Figure S7** (*a*) The asymmetry unit {[Eu_5_W_8_(H_2_O)_15_O_25_](B-α-AsW_9_O_33_)_4_}^23–^. (*b*) Two types of asymmetric heterometallic sandwich-type segments {Eu_2_(H_2_O)_6_W_3_O_10_[B-α-AsW_9_O_33_]_2_}^14–^ showing the mirror symmetry. (*c*) The dimeric [As_2_W_21_O_76_]^20–^ subunits generated from two {Eu_2_(H_2_O)_6_W_3_O_10_[B-α-AsW_9_O_33_]_2_}^14–^ units showing the mirror symmetry.

**Figure S8** (*a*) View of the symmetric unit {[Eu_10_W_16_(H_2_O)_30_O_50_](B-α-AsW_9_O_33_)_8_}^46–^. (*b*) View of the giant tungsten cluster with the omission of ten Eu^III^ centers along one direction. (*c*) View of the giant tungsten cluster with the omission of ten Eu^III^ centers along the other direction.

**Figure S9** The comparison of four kinds of typical {W_4_} groups.

**Figure S10** The 3-D packing structures of **1a** viewed along *a*, *b*, *c* axes.

**Figure S11** The UV spectra of **4** and **8**.

**Figure S12** (*a*) The UV spectral evolution of **8** with time. (*b*) The UV spectral evolution of **8** in acidic direction. (*c*) The UV spectral evolution of **8** in alkaline direction.

**Figure S13** (*a*) The UV spectrum of 0.3 mmol/L PBS (pH = 7.19). (*b*) The UV spectrum of **4** in 0.3 mmol/L PBS (pH = 7.08). (*c*) The UV spectrum evolution of **8** in 0.3 mmol/L PBS (pH = 7.13). (*d*) The UV spectrum of 0.3 mmol/L PBS containing 0.3% FBS (pH = 7.25). (*e*) The UV spectrum of **4** in 0.3 mmol/L PBS containing 0.3% FBS (pH = 7.23). (*f*) The UV spectrum of **8** in 0.3 mmol/L PBS containing 0.3% FBS (pH = 7.20).

**Figure S14** Morphological changes of MCF−7 cells incubated with (a) **4**, (b) **8** and (c) K_14_[As_2_W_19_O_67_(H_2_O)] with concentration of 1 mg/mL.

**Figure** **S15** IR spectra of **1–8**.

**Figure** **S16** The TG curves of **1**–**8**.

**Figure S17** The VTPXRD patterns of **3–6** displaying the similar variation trend.

**Figure S18** (a) The emission spectrum of **1** under excitation at 393 nm at room temperature. (b) The excitation spectrum of **1** obtained by monitoring the emission at 614 nm. (c) The luminescence decay curve of **1**. (d) The emission spectrum of **2** under excitation at 402 nm at room temperature. (e) The excitation spectrum of **2** obtained by monitoring the emission at 598 nm. (f) The luminescence decay curve of **2**. (g) The emission spectrum of **3** under excitation at 378 nm at room temperature. (h) The excitation spectrum of **3** obtained by monitoring the emission at 544 nm. (i) The luminescence decay curve of **3**. (j) The emission spectrum of **4** under excitation at 388 nm at room temperature. (k) The excitation spectrum of **4** obtained by monitoring the emission at 574 nm. (l) The luminescence decay curve of **4**.

**Figure S19** (a) The emission spectrum of K_14_[As_2_W_19_O_67_(H_2_O)] under excitation at 388 nm at room temperature. (b) The luminescence decay curve of K_14_[As_2_W_19_O_67_(H_2_O)].

**Figure S20** The CIE chromaticity diagram of the emissions of **1**, **2**, **4** and **5**.

**Table S1** The Eu–O bond lengths in **1**.

**Table S2** Half-maximal inhibitory concentrations (IC_50_) of **4**, **8** and K_14_[As_2_W_19_ O_67_(H_2_O)].

**Table** **S3** Crystal data and structural refinements for **1**–**8**.

**The photoluminescence properties of 1, 2, 4 and 5:**

Because Ln-based materials have the widespread potential applications in light emitting diodes, fluorescent tubes, sensory probes, tunable lasers and plasma display, etc,^1,2^ the solid state photoluminescence properties of **1**, **2**, **4** and **5** have been investigated in room temperature (Figure S18). Upon excitation at 393 nm, the photoluminescence spectrum of **1** exhibits five characteristic emission bands, which are attributed to ^5^D_0_→^7^F_0_ (580 nm), ^5^D_0_→^7^F_1_ (589 nm and 594 nm), ^5^D_0_→^7^F_2_ (614 nm and 618 nm), ^5^D_0_→^7^F_3_ (651 nm) and ^5^D_0_→^7^F_4_ (701nm) transitions of the Eu^III^ ions, respectively (Figure S18a).^3^ Generally, these transitions are hypersensitive to the change of the local environments around the Eu^III^ ions, and the intensity ratio of ^5^D_0_→^7^F_2_ (electric-dipole) / ^5^D_0_→^7^F_1_ (magnetic-dipole) is often used as a criterion to detect the local symmetry of the Eu^III^ ions.^4^ Specifically, the electronic-dipole ^5^D_0_→^7^F_2_ transition plays a leading role in a noncentrosymmetric chemical environment whereas the magnetic-dipole ^5^D_0_→^7^F_1_ transition is dominant in a centrosymmetric chemical environment.^5^ As for the as-synthesized **1**, the emission intensity ratio of ^5^D_0_→^7^F_2_/^5^D_0_→^7^F_1_ is 5.7, indicating that the lower site symmetry of the Eu^III^ ions in **1**, which coincides with the structure analysis results that the Eu^III^ ions adopt the eight-coordinate severely distorted square antiprism and seven-coordinate severely distorted monocapped trigonal prism geometries. The excitation spectrum of **1** has been obtained by monitoring the Eu^III^ ^5^D_0_→^7^F_2_ emission at 614 nm, which is dominated by a narrow line at 393 nm, corresponding to the ^7^F_0_→^5^L_6_ transition (Figure S18b).^5^ Moreover, the other excitation peaks (^7^F_0_→^5^D_4_, ^7^F_0_→^5^G_2_, ^7^F_0_→^5^D_3_) falling in 300–445 nm generated from the intra-4f transitions from the ^7^F_0_ ground state to higher energy levels of the Eu^3+^ ions are also seen.^6,7^ The ^5^D_0_ lifetime curve of the Eu^III^ ions has been also measured under the excitation light at 393 nm and the most intense emission at 614 nm (Figure S18c), which obeys the single-exponential function [*I* = *A*exp(−*t*/*τ*)], yielding the decay time of 269.12 μs, the pre-exponential factor (*A*) of 2463.11, and the agreement factor (*χ*^2^) of 1.202.

The emission spectrum of **2** under the excitation of visible light (402 nm) displays four characteristic luminescent bands, derived from the ^4^G_5/2_ excited state to lower ^6^H_J_ level transitions, which are ^4^G_5/2_→^6^H_5/2_ (561 nm), ^4^G_5/2_→^6^H_7/2_ (598 nm), ^4^G_5/2_ → ^6^H_9/2_ (644 nm) and ^4^G_5/2_ → ^6^H_11/2_ (705 nm) transitions, respectively (Figure S18d).^8^ The excitation spectrum of **2** obtained by monitoring the most intense peak of Sm^III^ ^4^G_5/2_→^6^H_7/2_ (598 nm) exhibits a broad peak originating from the ^6^H_5/2_→^4^K_11/2_ transition and several weak peaks derived from the ^6^H_5/2_→^4^K_17/2_, ^6^H_5/2_→^4^L_17/2_, ^6^H_5/2_→^4^F_7/2_ transitions of the Sm^III^ ions (Figure S18e).^9,10^ The lifetime curve of **2** has been also detected by monitoring the emission at 598 nm (^4^G_5/2_→^6^H_7/2_) and can be well fitted to a single-exponential function, generating the lifetime (*τ*) of 8.31 μs, the pre-exponential factor (*A*) of 856.79 and the agreement factor (*χ*^2^) of 1.184 (Figure S18f).

Figure S18g shows the emission spectrum of **4** under the excitation of the ultraviolet light (378 nm), in which four obvious luminescent emission bands with maxima at 489, 545, 582 and 622 nm can be assigned to ^5^D_4_→^7^F_6_, ^5^D_4_→^7^F_5_, ^5^D_4_→^7^F_4_ and ^5^D_4_→^7^F_3_ transitions of the Tb^III^ ions.^11^ The excitation spectrum of **4** has been collected by monitoring the most intense emission (^5^D_4_→^7^F_5_ transition) at 545 nm of the Tb^3+^ cations (Figure S18h), in which the strong excitation band at 378 nm is derived from the ^7^F_6_→^5^G_6_ transition of the Tb^III^ ions.^12^ The ^5^D_4_ lifetime curve of **4** has been under the excitation at 378 nm and the more intense emission 545 nm, which can also be fitted to a single-exponential function, affording the lifetime (*τ*) of 335.22 μs, the pre-exponential factor (*A*) of 1486.86, and the agreement factor (*χ*^2^) of 1.287 (Figure S18i).

In the case of **5**, the emission spectrum upon irradiation with the ultraviolet light of 388 nm consists of two characteristic emission bands with maxima at 480 and 574 nm, which are attributed to the ^4^F_9/2_→^6^H_15/2_ and ^4^F_9/2_→^6^H_13/2_ transitions of the Dy^3+^ ions (Figure S18j).^13^ The electric dipole transition ^4^F_9/2_→^6^H_13/2_ emission at 574 nm is stronger than the magnetic dipole transition ^4^F_9/2_→^6^H_15/2_ emission at 480 nm, which indicates that the Dy^III^ ions are located at the low symmetry environments.^14^ Monitoring the most strong Dy^III^ ^4^F_9/2_→^6^H_13/2_ transition at 547 nm, the excitation spectrum has been observed (Figure S18k) and demonstrates the ^6^H_15/2_→^6^P_7/2_, ^6^H_15/2_→^6^P_5/2_, ^6^H_15/2_→^4^I_13/2_ and ^6^H_15/2_→^4^G_11/2_ of the Dy^III^ ions, respectively.^15^ To further determine the lifetime, the luminescence decay curve of **5** was also carried out (Figure S18l). However, the decay curve of **5** can’t be fitted to a single exponential function, but a double exponential function *I* = *A*_1_ exp(−*t*/*τ*_1_) + *A*_2_ exp(−*t*/*τ*_2_) (where *τ*_1_ and *τ*_2_ are the fast and slow components of the luminescence lifetimes and *A*_1_ and *A*_2_ are the pre-exponential factors) affording the luminescence lifetimes *τ*_1_ and *τ*_2_ as 3.77 μs (36.15%) and 9.35 μs (63.85%), respectively. The average lifetime is about 7.33 μs. Furthermore, in order to probe why the decay time of **5** exhibits the second-order exponential function, the emission spectrum and the lifetime decay curve of K_14_[As_2_W_19_O_67_(H_2_O)] have also been investigated under similar conditions to **5** (Figure S19), the fitting values of τ_1_, τ_2_, A_1_ and A_2_ are 0.87 μs (37.19%), 9.45 μs (62.81%), 5180.76 and 806.82, respectively and the average lifetime is 6.26 μs. These results indicate that the arsentungstate fragments have a significant contribution to the lifetime of **5**. Therefore, the emission behavior of **5** can be generated from the combined action of the characteristic ^4^F_9/2_→^6^H_J_ (*J* = 15/2 and 13/2) transitions of the Dy^III^ ions and the O→W LMCT transitions. Thus, it can be presumed that the large contribution of the O→W LMCT transitions during the emission process of **5** results in the case that the decay curve of **5** confirms to the second-order exponential function.

Based on the corresponding emission spectra, the CIE chromaticity coordinates of **1**, **2**, **4** and **5** are indexed to (0.64858, 0.35086), (0.60025, 0.39765), (0.33161, 0.60314) and (0.38868, 0.41326) (Figure S20), which display the red, reddish orange, green and yellow-green emissions, respectively.

1. Cui, Y. J., Yue, Y. F., Qian, G. D. & Chen, B. L. Luminescent functional metal–organic frameworks. *Chem. Rev.* **112**, 1126–1162 (2012).
2. Ma, X., Yang, W., Chen, L. J. & Zhao, J. W. Significant developments in rare-earth-containing polyoxometalate chemistry: synthetic strategies, structural diversities and correlative properties. *CrystEngComm* **17**, 8175–8197 (2015).
3. Xia, J., *et al*. Two- and three-dimensional lanthanide complexes: synthesis, crystal structures, and properties. *Inorg. Chem.* **46**, 3450–3453 (2007).
4. Li, H. L., *et al*. Self-assembly of a family of isopolytungstates induced by the synergistic effect of the nature of lanthanoids and the pH variation in the reaction process: syntheses, structures, and properties. *Cryst. Growth Des*. **16**, 108−120 (2016).
5. Hungerford, G., Suhling, K., & Green, M. Luminescence enhancement of a europium containing polyoxometalate on interaction with bovine serum albumin. *Photochem. Photobiol. Sci.* **7**, 734-737 (2008).
6. Zhang, Q. J., *et al*. Enhancing the photoluminescence intensity of CaTiO_3_:Eu^3+^ red phosphors with magnesium. *J. Rare Earth.* **33**, 1036–1039 (2015).
7. Hou, J. S., Yin, X., Huang, F. Q. & Jiang, W. Z. Synthesis and photoluminescence properties of NaLaMgWO_6_:RE^3+^ (RE = Eu, Sm, Tb) phosphor for white LED application. *Mater. Res. Bull.* **47**, 1295–1300 (2012).
8. Huang, Y.-G., *et al*. New lanthanide hybrid as clustered infinite nanotunnel with 3D Ln−O−Ln framework and (3,4)-connected net, *Inorg. Chem.* **46**, 1171–1176 (2007).
9. Zhang, Z. J., *et al*. Photoluminescence properties and energy level locations of RE^3+^ (RE = Pr, Sm, Tb, Tb/Ce) in CaAlSiN_3_ phosphors. *J. Mater. Chem.* **22**, 9813–9820 (2012).
10. Yang, Z. P., *et al*. Photoluminescence properties of Sm^3+^-doped LiY(MoO_4_)_2_ red phosphors. *J. Rare Earth.* **32**, 404–408 (2014).
11. Chorazy, S., Nakabayashi, K., Ohkoshi, S.-I. & Sieklucka, B. Green to red luminescence switchable by excitation light in cyanido-bridged Tb^III^–W^V^ ferromagnet. *Chem. Mater*. **26**, 4072–4075 (2014).
12. Li, X.; Sun, H.-L.; Wu, X.-S.; Qiu, X.; Du, M. Unique (3,12)-connected porous lanthanide−organic frameworks based on Ln_4_O_4_ clusters: synthesis, crystal structures, luminescence, and magnetism. *Inorg. Chem.* **49**, 1865–1871 (2010).
13. Zhang, Y., *et al*. A new single-phase white-light-emitting CaWO_4_:Dy^3+^ phosphor: synthesis, luminescence and energy transfer, *RSC Adv.* **5**, 62527–62533 (2015).
14. Wu, L., *et al*. Luminescence and energy transfer of a color tunable phosphor: Dy^3+^-, Tm^3+^-, and Eu^3+^- coactivated KSr_4_(BO_3_)_3_ for warm white UV LEDs. *J. Mater. Chem.* **22**, 6463–6470 (2012).
15. Deng, Y. M., Yi, S. P., Wang, Y. H. & Xian, J. Q. Synthesis and photoluminescence characteristics of Ln^3+^ (Ln = Sm, Er and Dy)-doped BaGd_2_(MoO_4_)_4_ phosphors. *Opt. Mater.* **36**, 1378–1383 (2014).


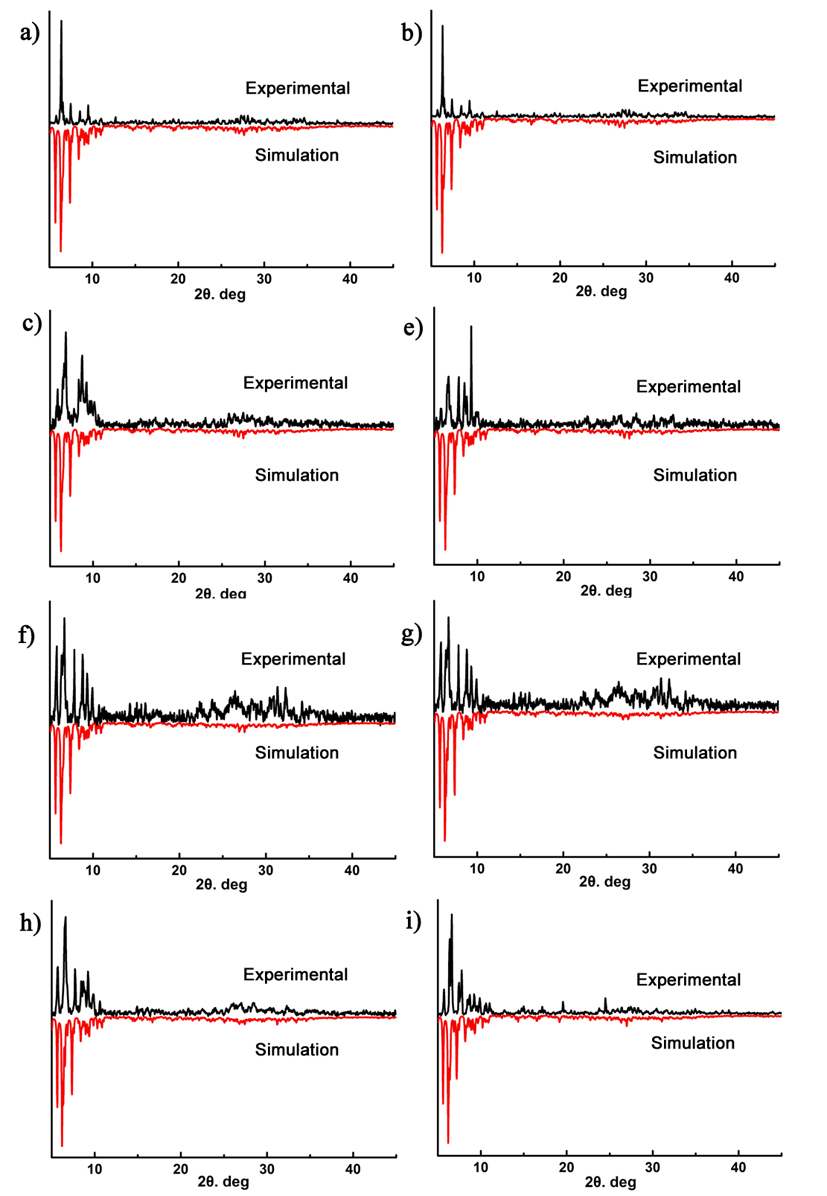


**Figure** **S1** (a) The experimental PXRD and simulated XRD patterns of the as-prepared sample of **1**. (b) The experimental PXRD and simulated XRD patterns of the as-prepared sample of **2**. (c) The experimental PXRD and simulated XRD patterns of the as-prepared sample of **3**. (d) The experimental PXRD and simulated XRD patterns of the as-prepared sample of **4**. (e) The experimental PXRD and simulated XRD patterns of the as-prepared sample of **5**. (f) The experimental PXRD and simulated XRD patterns of the as-prepared sample of **6**. (g) The experimental PXRD and simulated XRD patterns of the as-prepared sample of 7. (h) The experimental PXRD and simulated XRD patterns of the as-prepared sample of **8**.


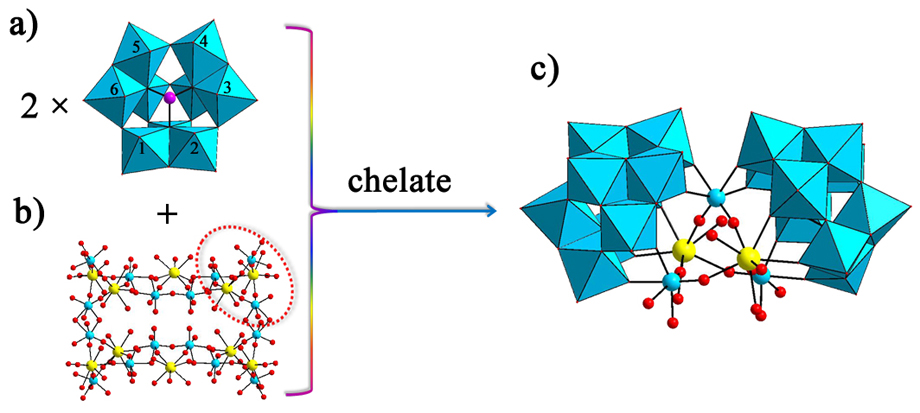


**Figure S2** (*a*) The inorganic multi-dentate [B-α-AsW_9_O_33_]^9–^ ligand. (*b*) The rectangular [Eu_10_W_16_(H_2_O)_30_O_50_]^26+^ cluster core. (*c*) The representative chelating mode between two inorganic multi-dentate [B-α-AsW_9_O_33_]^9–^ ligands and the center core.


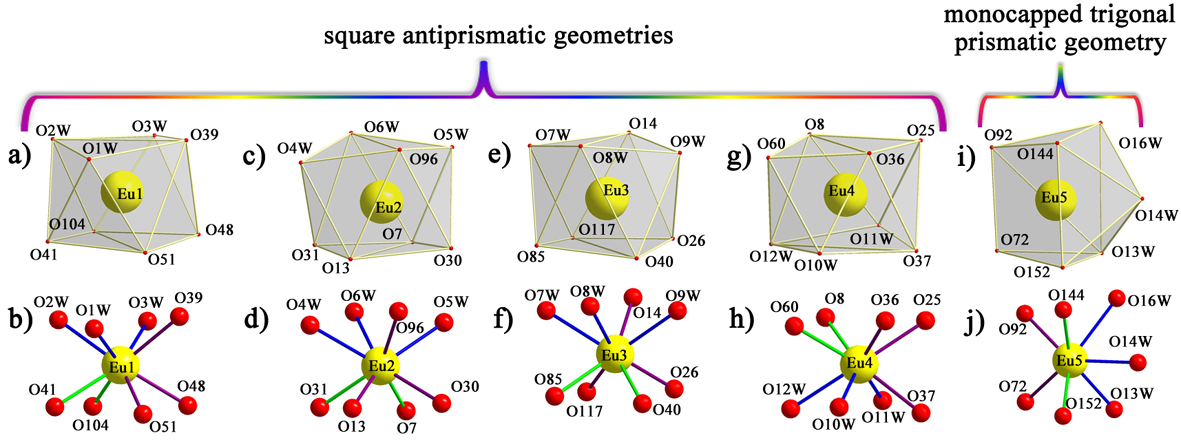


**Figure S3** (*a*-*h*) The eight-coordinate square antiprism geometries of Eu^III^ cations in the {W_3_Eu_2_} segments with the highlighted different sources of oxygen atoms. (*i*-*j*) The seven-coordinate monocapped trigonal prism geometry of the Eu^III^ cation in the {W_2_Eu} segment with the highlighted different sources of oxygen atoms. (The blue bonds link the oxygen atoms from water ligands, the green bonds link the bridging oxygen atoms from [B-α-AsW_9_O_33_]^9–^ units and the purple bonds link the oxygen atoms from other the octahedral W centers)


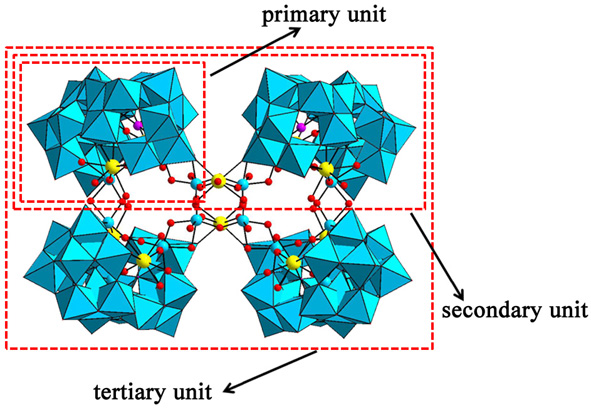


**Figure S4** The structural disassembly of **1a** in which the primary unit is the pentanuclear heterometallic sandwich-type unit {Eu_2_(H_2_O)_6_W_3_O_10_[B-α-AsW_9_O_33_]_2_}^14–^, the secondary unit is the asymmetric unit {[Eu_5_W_8_(H_2_O)_15_O_25_](B-α-AsW_9_O_33_)_4_}^23–^ and the tertiary unit is the molecular structural unit {[Eu_10_W_16_(H_2_O)_30_O_50_](B-α-AsW_9_O_33_)_8_}^46–^.


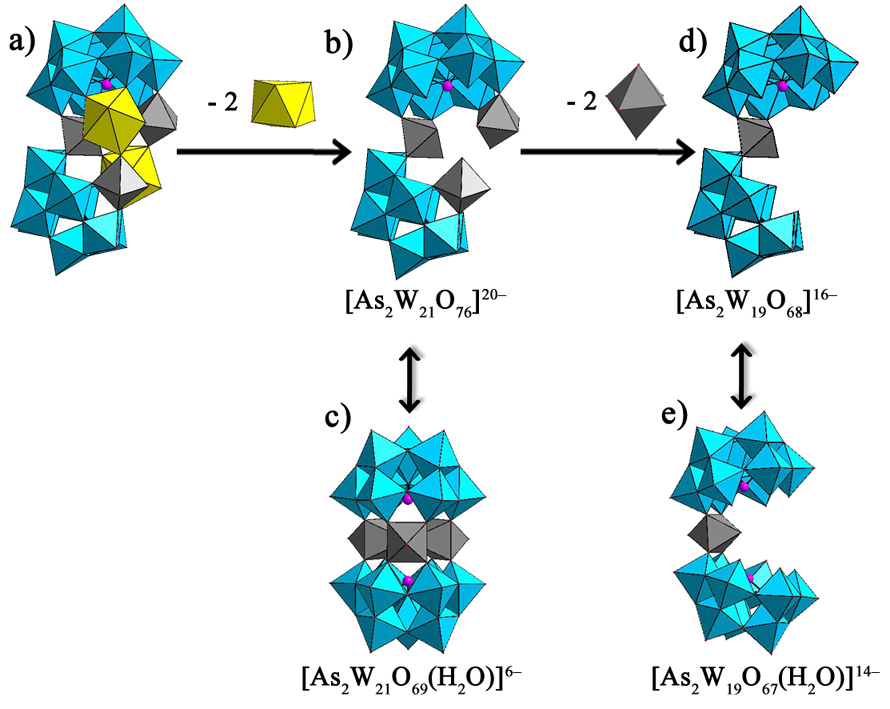


**Figure S5** (*a*) The skeleton of the primary unit {Eu_2_(H_2_O)_6_W_3_O_10_[B-α-AsW_9_O_33_]_2_}^14–^. (*b*) View of the interesting dimeric [As_2_W_21_O_76_]^20–^ unit from the primary unit. (*c*) The structure of the previously reported [As_2_W_21_O_69_(H_2_O)]^6–^ precursor. (*d*) The distorted [As_2_W_19_O_68_]^16–^ fragment. (*e*) The [As_2_W_19_O_67_(H_2_O)]^14–^ polyoxoanion.


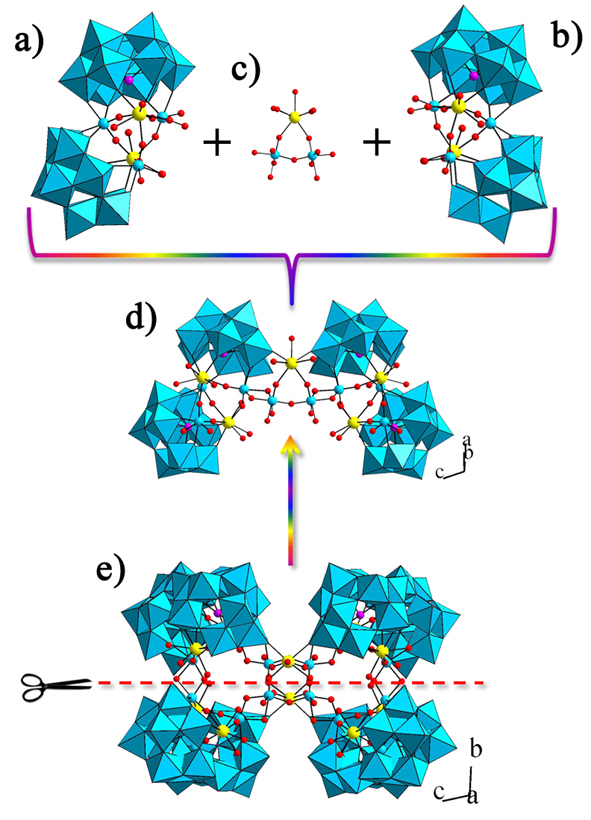


**Figure** **S6** (*a*) and (*b*) The skeleton of the pentanuclear heterometallic sandwich-type unit {Eu_2_(H_2_O)_6_W_3_O_10_[B-α-AsW_9_ O_33_]_2_}^14–^. (*c*) The bridging {W_2_Eu_1_} segment. (*d*) View of the asymmetric unit {[Eu_5_W_8_(H_2_O)_15_O_25_](B-α-AsW_9_O_33_)_4_}^23–^. (*e*) The skeleton of the symmetric unit {[Eu_10_W_16_(H_2_O)_30_ O_50_](B-α-AsW_9_O_33_)_8_}^46–^.


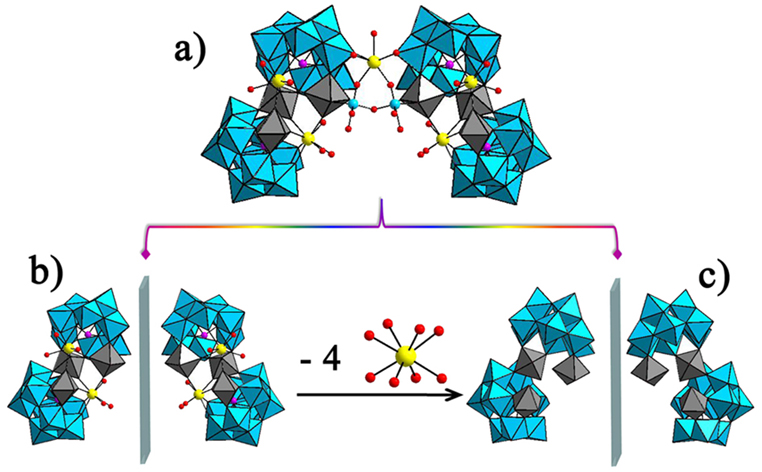


**Figure S7** (*a*) The asymmetry unit {[Eu_5_W_8_(H_2_O)_15_O_25_](B-α-AsW_9_O_33_)_4_}^23–^. (*b*) Two types of asymmetric heterometallic sandwich-type segments {Eu_2_(H_2_O)_6_W_3_O_10_[B-α-AsW_9_O_33_]_2_}^14–^ showing the mirror symmetry. (*c*) The dimeric [As_2_W_21_O_76_]^20–^ subunits generated from two {Eu_2_(H_2_O)_6_W_3_O_10_[B-α-AsW_9_O_33_]_2_}^14–^ units showing the mirror symmetry.


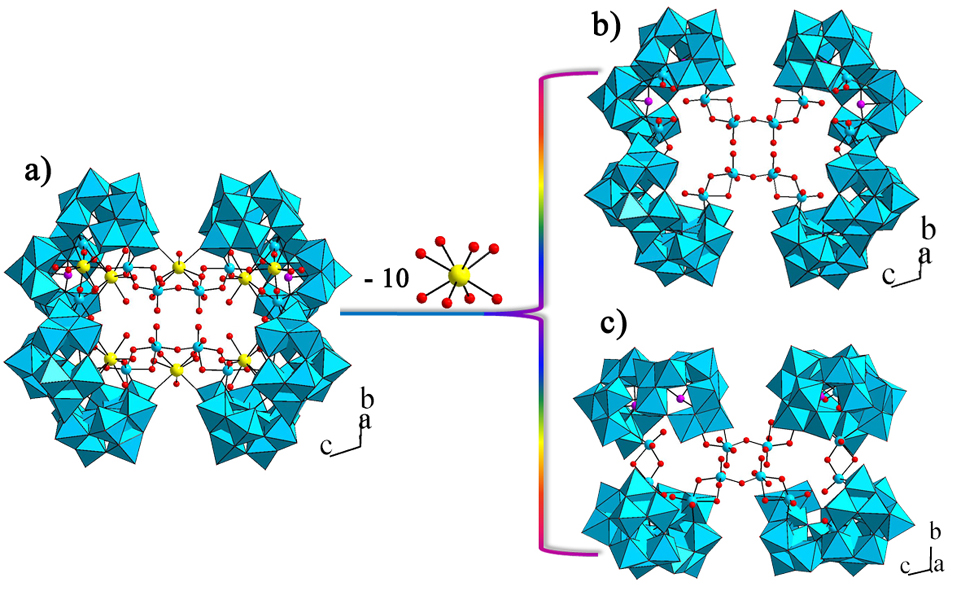


**Figure S8** (*a*) View of the symmetric unit {[Eu_10_W_16_(H_2_O)_30_O_50_](B-α-AsW_9_O_33_)_8_}^46–^. (*b*) View of the giant tungsten cluster with the omission of ten Eu^III^ centers along one direction. (*c*) View of the giant tungsten cluster with the omission of ten Eu^III^ centers along the other direction.


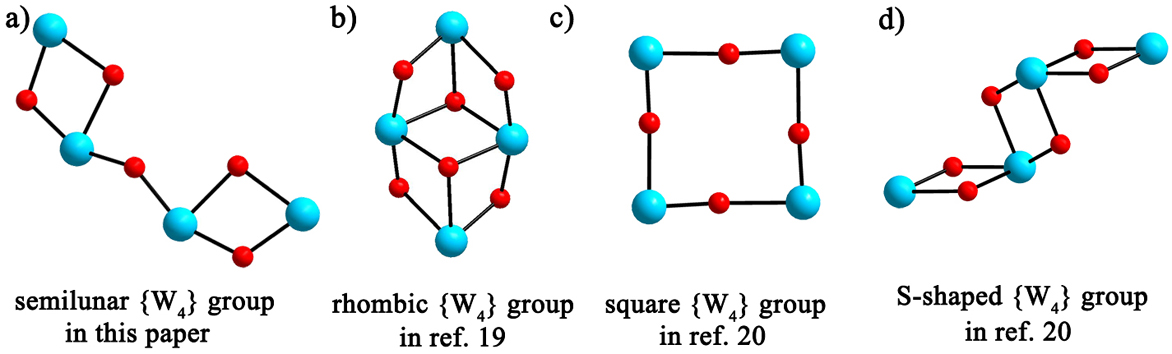


**Figure S9** The comparison of four kinds of typical {W_4_} groups.


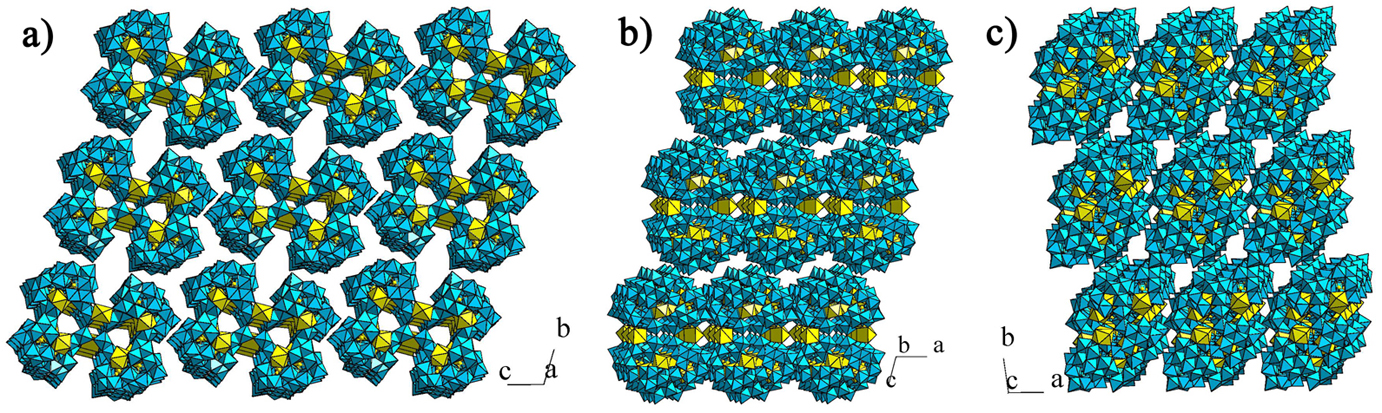


**Figure S10** The 3-D packing structures of **1a** viewed along *a*, *b*, *c* axes.


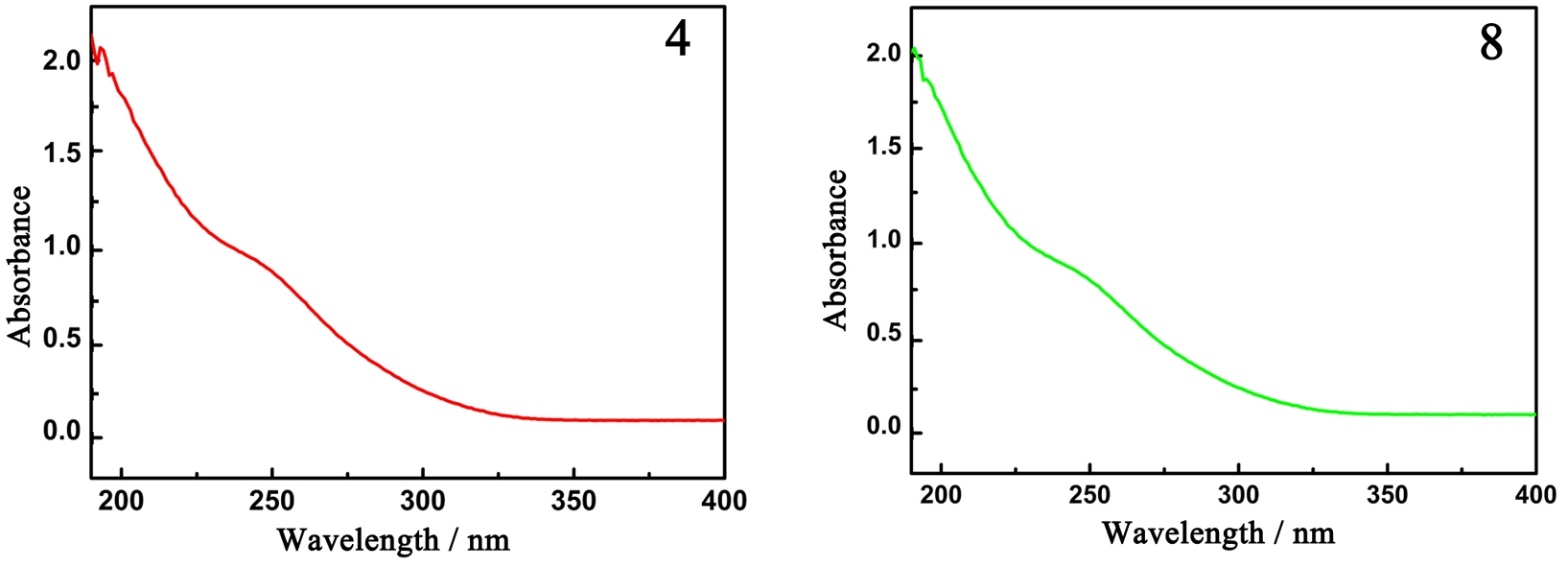


**Figure S11** The UV spectra of **4** and **8**.


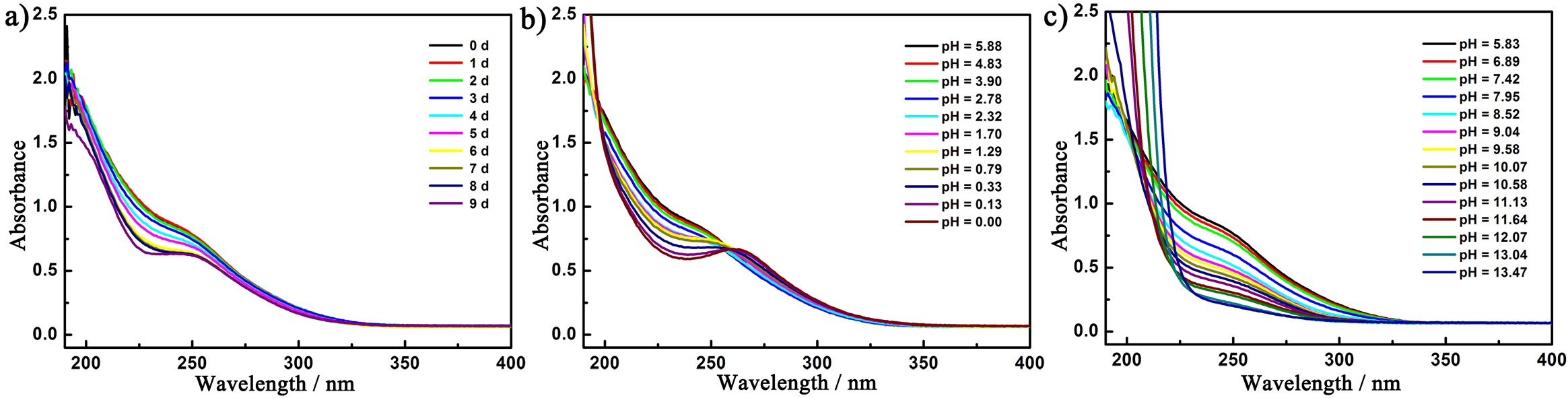


**Figure S12** (*a*) The UV spectral evolution of **8** with time. (*b*) The UV spectral evolution of **8** in acidic direction. (*c*) The UV spectral evolution of **8** in alkaline direction.


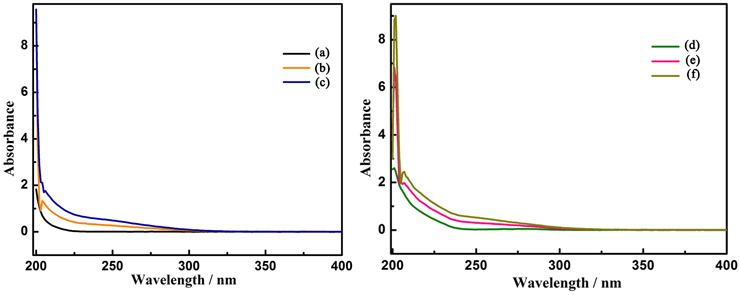


**Figure S13** (*a*) The UV spectrum of 0.3 mmol/L PBS (pH = 7.19). (*b*) The UV spectrum of **4** in 0.3 mmol/L PBS (pH = 7.08). (*c*) The UV spectrum evolution of **8** in 0.3 mmol/L PBS (pH = 7.13). (*d*) The UV spectrum of 0.3 mmol/L PBS containing 0.3% FBS (pH = 7.25). (*e*) The UV spectrum of **4** in 0.3 mmol/L PBS containing 0.3% FBS (pH = 7.23). (*f*) The UV spectrum of **8** in 0.3 mmol/L PBS containing 0.3% FBS (pH = 7.20).


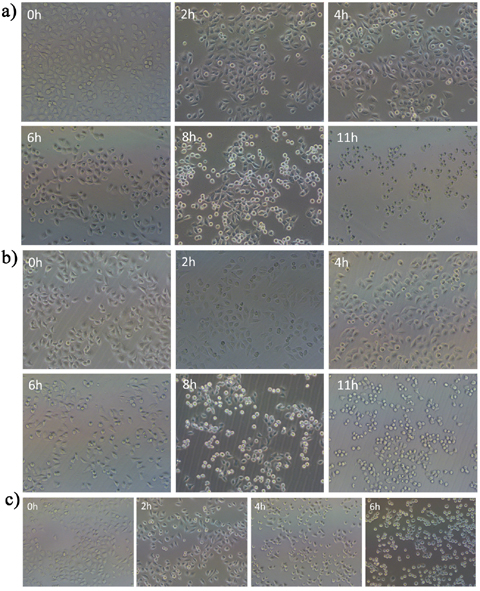


**Figure S14** Morphological changes of MCF−7 cells incubated with (a) **4**, (b) **8** and (c) K_14_[As_2_W_19_O_67_(H_2_O)] with concentration of 1 mg/mL.


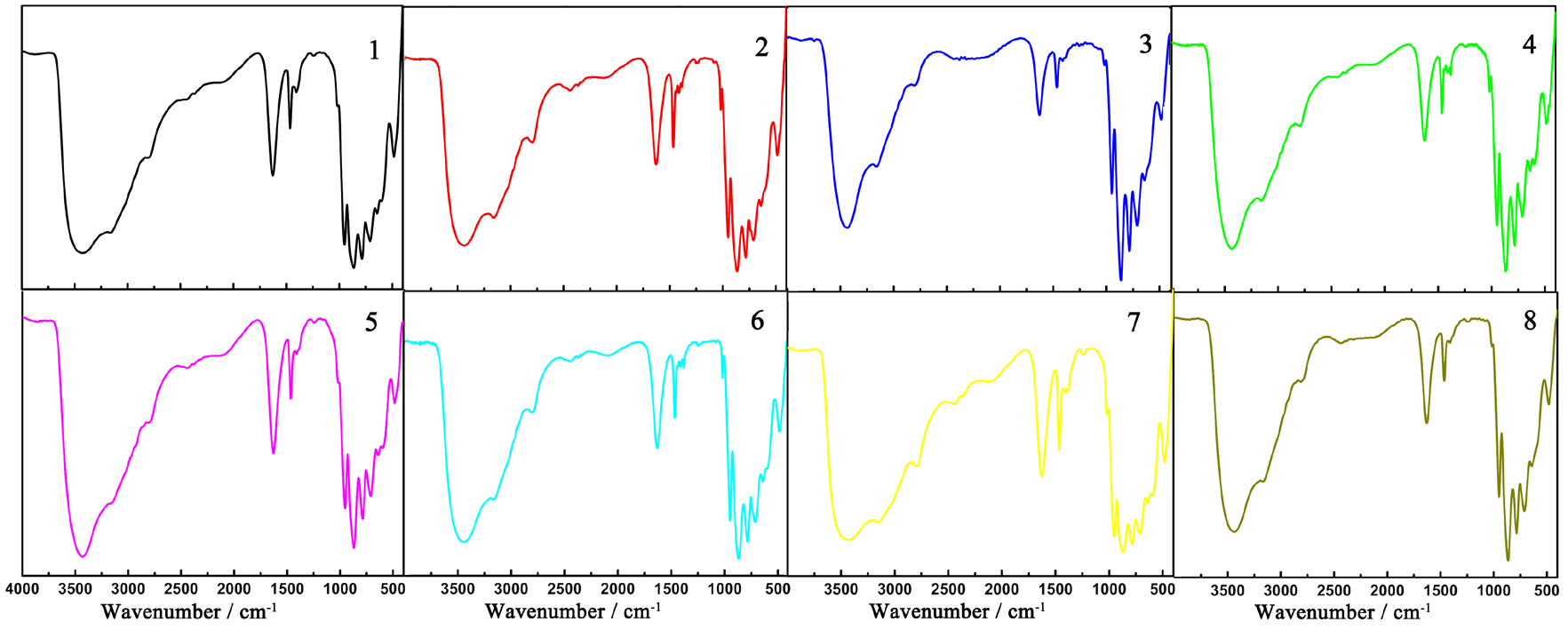


**Figure S15** IR spectra of **1–8**.


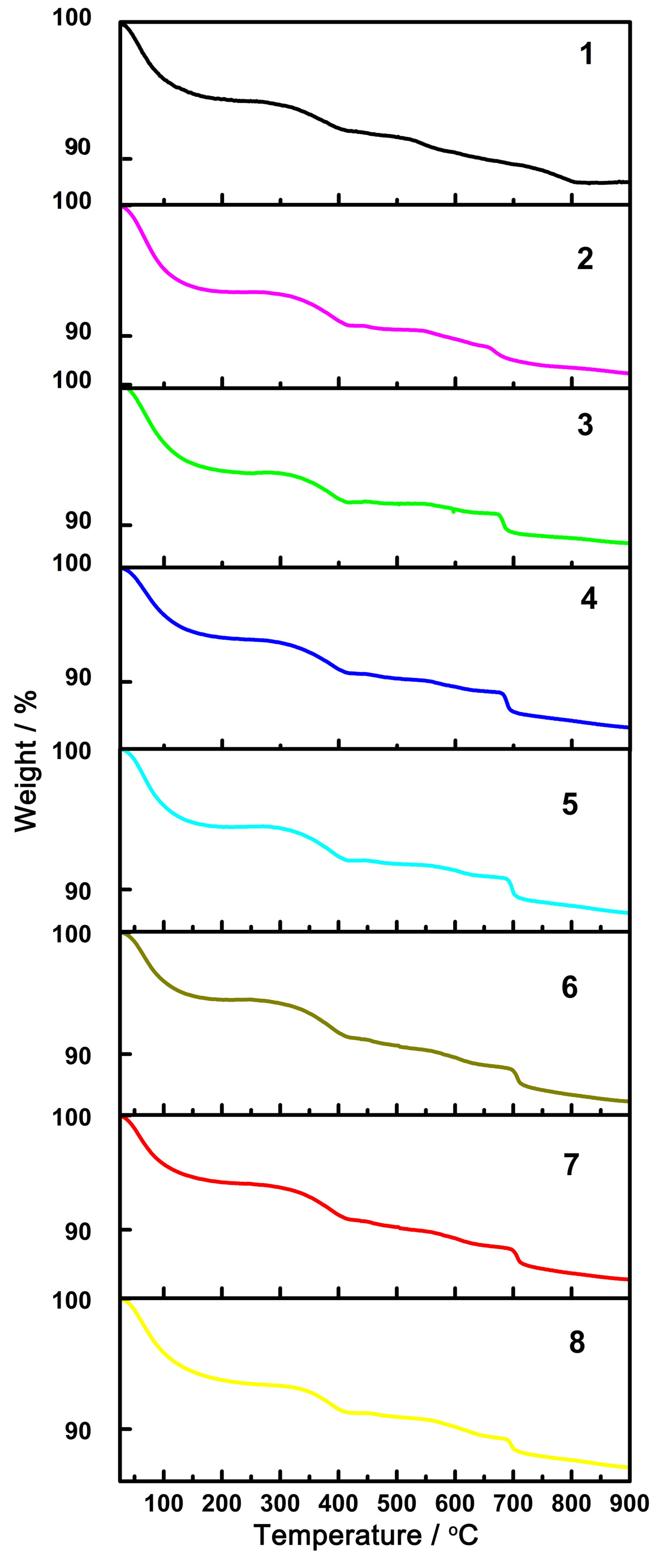


**Figure S16** The TG curves of **1**–**8**.


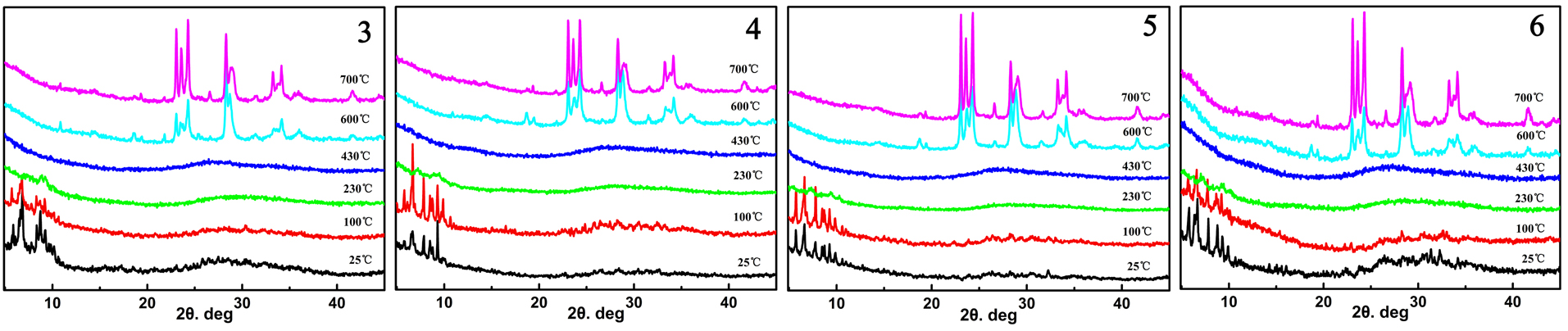


**Figure S17** The VTPXRD patterns of **3–6** displaying the similar variation trend.


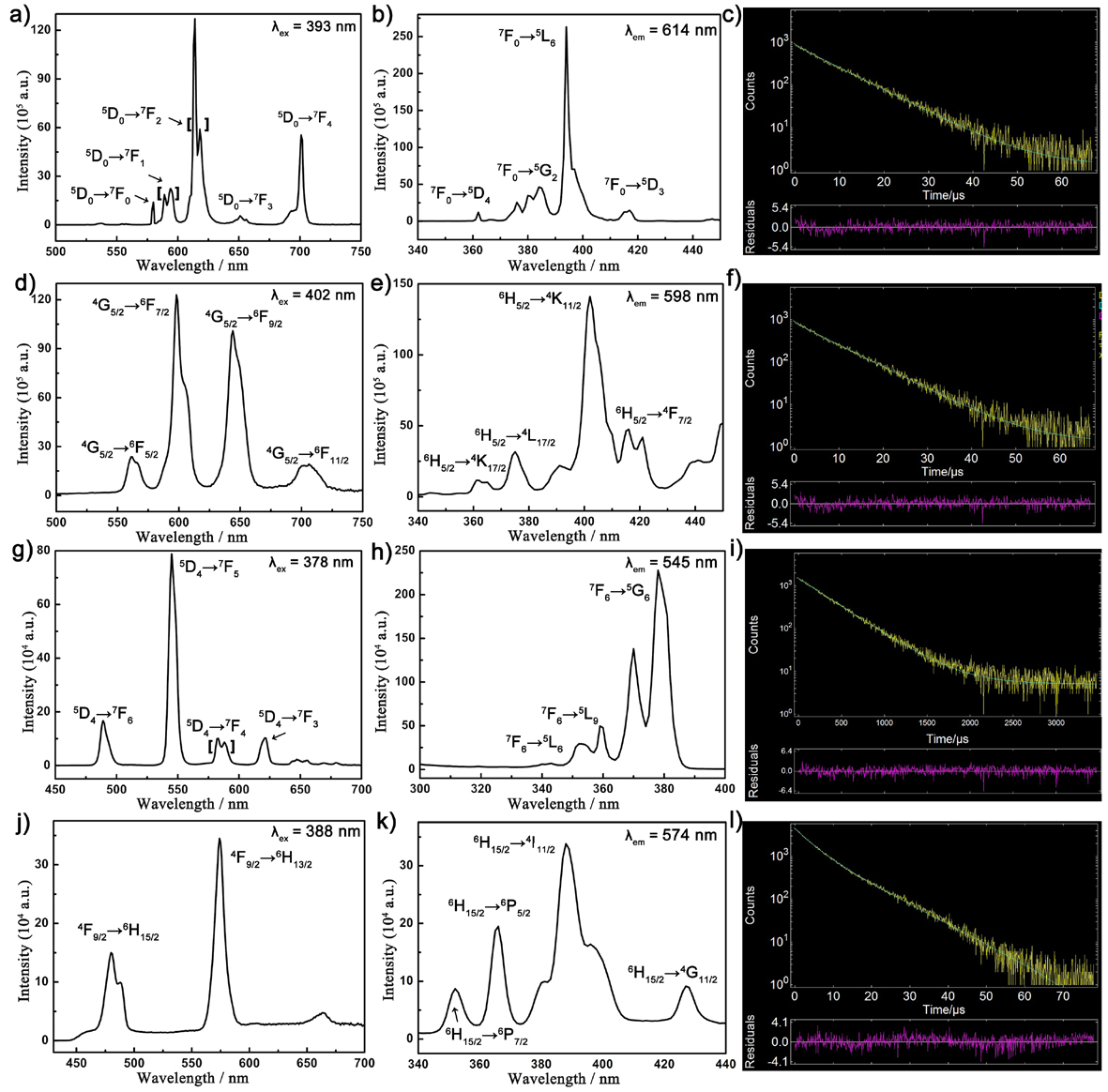


**Figure S18** (a) The emission spectrum of **1** under excitation at 393 nm at room temperature. (b) The excitation spectrum of **1** obtained by monitoring the emission at 614 nm. (c) The luminescence decay curve of **1**. (d) The emission spectrum of **2** under excitation at 402 nm at room temperature. (e) The excitation spectrum of **2** obtained by monitoring the emission at 598 nm. (f) The luminescence decay curve of **2**. (g) The emission spectrum of **4** under excitation at 378 nm at room temperature. (h) The excitation spectrum of **4** obtained by monitoring the emission at 544 nm. (i) The luminescence decay curve of **4**. (j) The emission spectrum of **5** under excitation at 388 nm at room temperature. (k) The excitation spectrum of **5** obtained by monitoring the emission at 574 nm. (l) The luminescence decay curve of **5**.


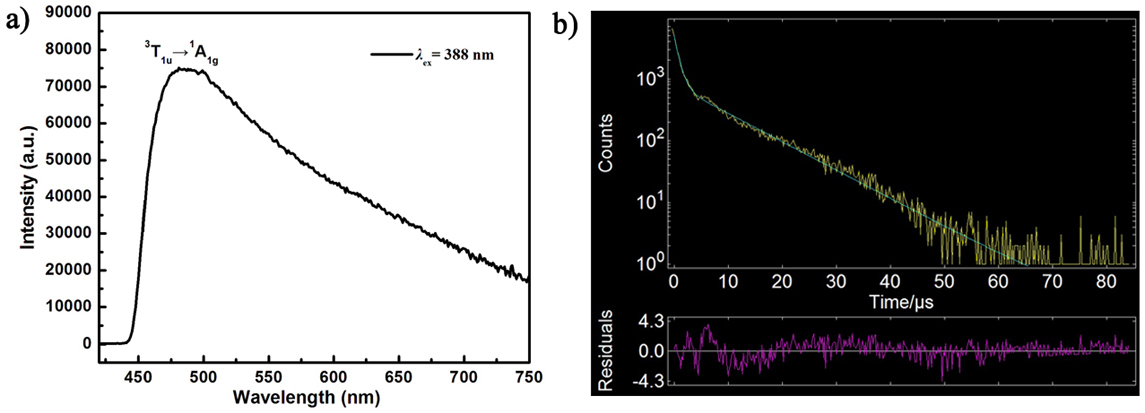


**Figure S19** (a) The emission spectrum of K_14_[As_2_W_19_O_67_(H_2_O)] under excitation at 388 nm at room temperature. (b) The luminescence decay curve of K_14_[As_2_W_19_O_67_(H_2_O)].


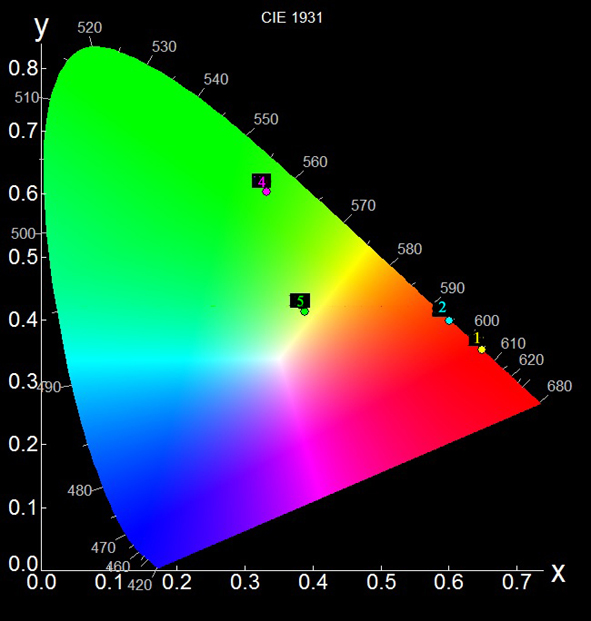


**Figure S20** The CIE chromaticity diagram of the emissions of **1**, **2**, **4** and **5**.

**Table S1** The Eu–O bond lengths in **1**.

| Bond | Length / Å | Bond | Length / Å |
| --- | --- | --- | --- |
| Eu(1)–O(51) | 2.284(16) | Eu(3)–O(26) | 2.393(17) |
| Eu(1)–O(104) | 2.369(17) | Eu(3)–O(9W) | 2.426(18) |
| Eu(1)–O(2W) | 2.382(18) | Eu(3)–O(8W) | 2.437(18) |
| Eu(1)–O(48) | 2.389(16) | Eu(3)–O(7W) | 2.491(19) |
| Eu(1)–O(39) | 2.407(15) | Eu(4)–O(36) | 2.311(16) |
| Eu(1)–O(41) | 2.410(17) | Eu(4)–O(37) | 2.379(15) |
| Eu(1)–O(3W) | 2.446(19) | Eu(4)–O(8) | 2.394(15) |
| Eu(1)–O(1W) | 2.497(19) | Eu(4)–O(60) | 2.400(15) |
| Eu(2)–O(13) | 2.287(14) | Eu(4)–O(25) | 2.408(16) |
| Eu(2)–O(7) | 2.328(13) | Eu(4)–O(10W) | 2.429(17) |
| Eu(2)–O(31) | 2.360(17) | Eu(4)–O(11W) | 2.474(18) |
| Eu(2)–O(96) | 2.400(16) | Eu(4)–O(12W) | 2.489(18) |
| Eu(2)–O(30) | 2.411(15) | Eu(5)–O(152) | 2.33(2) |
| Eu(2)–O(5W) | 2.423(17) | Eu(5)–O(144) | 2.375(17) |
| Eu(2)–O(6W) | 2.498(18) | Eu(5)–O(13W) | 2.40(2) |
| Eu(2)–O(4W) | 2.527(16) | Eu(5)–O(92) | 2.404(16) |
| Eu(3)–O(117) | 2.304(14) | Eu(5)–O(72) | 2.406(15) |
| Eu(3)–O(85) | 2.357(18) | Eu(5)–O(14W) | 2.43(2) |
| Eu(3)–O(14) | 2.372(15) | Eu(5)–O(16W) | 2.57(3) |
| Eu(3)–O(40) | 2.390(16) |  |  |

**Table S2**. Half-maximal inhibitory concentrations (IC_50_) of **4**, **8** and K_14_[As_2_W_19_ O_67_(H_2_O)].

| Compound | IC_50_ (μg/mL) | | |
| --- | --- | --- | --- |
|  | HeLa | MCF–7 | L929 |
| **4** | 40.05 | 40.32 | 59.68 |
| **8** | 24.76 | 37.01 | 58.04 |
| K_14_[As_2_W_19_O_67_(H_2_O)] | 27.56 | 32.00 | – |

**Table S3** Crystal data and structural refinements for **1**–**8**.

|  | **1** | **2** | **3** | **4** |
| --- | --- | --- | --- | --- |
| Empirical formula | C_12_H_318_As_8_Eu_10_N_6_ Na_24_ O_441_W_88_ | C_12_H_318_As_8_Sm_10_N_6_ Na_24_O_441_W_88_ | C_12_H_318_As_8_Gd_10_N_6_ Na_24_O_441_W_88_ | C_12_H_318_As_8_Tb_10_N_6_ Na_24_O_441_W_88_ |
| Fw | 26454.24 | 26438.14 | 26507.14 | 26523.84 |
| Crystal system | triclinic | triclinic | triclinic | triclinic |
| Space group | *P*–1 | *P*–1 | *P*–1 | *P*–1 |
| *a*, Å | 18.8558(16) | 18.9984(9) | 19.0101(17) | 18.940(4) |
| *b,* Å | 24.244(2) | 24.3919(12) | 24.407(2) | 24.333(5) |
| *c,* Å | 29.824(3) | 29.9953(15) | 30.023(3) | 29.920(6) |
| *α,* deg | 104.990(2) | 104.7320(10) | 104.652(2) | 104.699(4) |
| *β,* deg | 102.128(2) | 101.6460 | 101.775(2) | 101.781(4) |
| *γ,* deg | 95.176(2) | 95.2510(10) | 95.352(2) | 95.444(4) |
| *V ,* Å^-3^ | 12724.2(19) | 13015.7(11) | 13038(2) | 12900(4) |
| *Z* | 1 | 1 | 1 | 1 |
| μ, mm^–1^ | 21.645 | 21.083 | 21.193 | 21.505 |
| *F*(000) | 11630 | 11620 | 11640 | 11650 |
| *T*, K | 296(2) | 296(2) | 296(2) | 296(2) |
| Limiting indices | –22 ≤ *h* ≤ 22  –28 ≤ *k* ≤ 19  –35 ≤ *l* ≤ 35 | –22 ≤ *h* ≤ 22  –29 ≤ *k* ≤ 20  –34 ≤ *l* ≤ 35 | –22 ≤ *h* ≤ 22  –29 ≤ *k* ≤ 25  –34 ≤ *l* ≤ 35 | –22 ≤ *h* ≤ 22  –21 ≤ *k* ≤ 28  –34 ≤ *l* ≤ 35 |
| No. of reflections collected | 65974 | 67648 | 67531 | 65110 |
| No. of independent reflections | 44526 | 45579 | 45659 | 44879 |
| *R*_int_ | 0.0640 | 0.0636 | 0.0711 | 0.1055 |
| Data / restrains / parameters | 44526 / 117 / 2134 | 45579 / 151 / 2125 | 45659 / 169 / 2127 | 44879 / 653 / 2072 |
| Goodness-of-fit on *F*^2^ | 1.034 | 1.028 | 1.000 | 1.034 |
| Final *R* indices [*I*>2*σ*(*I*)] | *R*_1_ = 0.0679  *wR*_2_ = 0.1503 | *R*_1_ = 0.0646,  *wR*_2_ = 0.1354 | *R*_1_ = 0.0673  *wR*_2_ = 0.1450 | *R*_1_ = 0.1515  *wR*_2_ = 0.3596 |
| *R* indices (all data) | *R*_1_ = 0.1297  *wR*_2_ = 0.1685 | *R*_1_ = 0.1395  *wR*_2_ = 0.1543 | *R*_1_ = 0.1370  *wR*_2_ = 0.1631 | *R*_1_ = 0.2213  *wR*_2_ = 0.3868 |

**Table S3** Continued.

|  | **5** | **6** | **7** | **8** |
| --- | --- | --- | --- | --- |
| Empirical formula | C_12_H_318_As_8_Dy_10_N_6_ Na_24_O_441_W_88_ | C_12_H_318_As_8_Ho_10_N_6_Na_24_O_441_W_88_ | C_12_H_318_As_8_Er_10_N_6_ Na_24_O_441_W_88_ | C_12_H_318_As_8_Tm_10_N_6_Na_24_O_441_W_88_ |
| Fw | 26559.64 | 26583.95 | 26607.24 | 26623.95 |
| Crystal system | triclinic | triclinic | triclinic | triclinic |
| Space group | *P*–1 | *P*–1 | *P*–1 | *P*–1 |
| *a*, Å | 18.992(2) | 18.996(2) | 19.070(3) | 19.147(18) |
| *b,* Å | 24.363(3) | 24.282(3) | 24.337(4) | 24.88(2) |
| *c,* Å | 29.992(4) | 30.143(4) | 30.332(5) | 30.49(3) |
| *α,* deg | 104.537(3) | 104.462(2) | 104.248(4) | 105.661(16) |
| *β,* deg | 101.969(3) | 102.429(2) | 102.652(4) | 101.977(16) |
| *γ,* deg | 95.397(3) | 95.195(2) | 95.160(4) | 94.802(16) |
| *V ,* Å^-3^ | 12983(3) | 12994(3) | 13159(4) | 13529(21) |
| *Z* | 1 | 1 | 1 | 1 |
| μ, mm^–1^ | 21.444 | 21.512 | 21.333 | 20.837 |
| *F*(000) | 11660 | 11670 | 11680 | 11690 |
| *T*, K | 296(2) | 296(2) | 296(2) | 296(2) |
| Limiting indices | –22 ≤ *h* ≤ 22  –28 ≤ *k* ≤ 28  –35 ≤ *l* ≤ 34 | –22 ≤ *h* ≤ 22  –28 ≤ *k* ≤ 24  –35 ≤ *l* ≤ 34 | –22 ≤ *h* ≤ 22  –23 ≤ *k* ≤ 28  –36 ≤ *l* ≤ 31 | –22 ≤ *h* ≤ 22  –29 ≤ *k* ≤ 29  –36 ≤ *l* ≤ 33 |
| No. of reflections collected | 67053 | 66271 | 67311 | 65395 |
| No. of independent reflections | 45366 | 45159 | 45865 | 45949 |
| *R*_int_ | 0.0963 | 0.0977 | 0.1129 | 0.0994 |
| Data / restrains / parameters | 45366 / 388 / 2069 | 45159 / 692 / 2101 | 45865 / 626 / 2086 | 45949 / 527 / 1852 |
| Goodness-of-fit on *F*^2^ | 1.016 | 1.029 | 0.997 | 1.072 |
| Final *R* indices [*I*>2*σ*(*I*)] | *R*_1_ = 0.0879  *wR*_2_ = 0.1695 | *R*_1_ = 0.1086  *wR*_2_ = 0.2575 | *R*_1_ = 0.0695  *wR*_2_ = 0.1234 | *R*_1_ = 0.1241  *wR*_2_ = 0.3111 |
| *R* indices (all data) | *R*_1_ = 0.2076  *wR*_2_ = 0.1948 | *R*_1_ = 0.2059  *wR*_2_ = 0.2905 | *R*_1_ = 0.2133  *wR*_2_ = 0.1420 | *R*_1_ = 0.2138  *wR*_2_ = 0.3533 |
